# Association of Obesity With Survival Outcomes in Patients With Cancer: A Systematic Review and Meta-analysis

**DOI:** 10.1001/jamanetworkopen.2021.3520

**Published:** 2021-03-29

**Authors:** Fausto Petrelli, Alessio Cortellini, Alice Indini, Gianluca Tomasello, Michele Ghidini, Olga Nigro, Massimiliano Salati, Lorenzo Dottorini, Alessandro Iaculli, Antonio Varricchio, Valentina Rampulla, Sandro Barni, Mary Cabiddu, Antonio Bossi, Antonio Ghidini, Alberto Zaniboni

**Affiliations:** 1 Oncology Unit, Azienda Socio Sanitaria Territoriale Bergamo Ovest, Treviglio, Italy; 2 Oncology Unit, Department of Biotechnology and Applied Clinical Sciences, San Salvatore Hospital, University of L’Aquila, L’Aquila, Italy; 3 Oncology Unit, Fondazione Istituto di Ricovero e Cura a Carattere Scientifico Ospedale Maggiore Policlinico, Milano, Italy; 4 Oncology Unit, Azienda Socio Sanitaria Territoriale Sette Laghi, Ospedale di Circolo, Varese, Italy; 5 Oncology Unit, University Hospital of Modena, Modena Cancer Centre, Modena, Italy; 6 Oncology Unit, Azienda Socio Sanitaria Territoriale Bergamo Est, Seriate, Italy; 7 Surgical Oncology Unit, Azienda Socio Sanitaria Territoriale Bergamo Ovest, Treviglio, Italy; 8 Endocrine Diseases Unit–Diabetes Regional Center, Azienda Socio Sanitaria Territoriale Bergamo Ovest, Treviglio, Italia; 9 Oncology Unit, Casa di Cura Igea, Milano, Italy; 10 Oncology Unit, Fondazione Poliambulanza, Brescia, Italy

## Abstract

**Importance:**

Obesity, defined as a body mass index (BMI) greater than 30, is associated with a significant increase in the risk of many cancers and in overall mortality. However, various studies have suggested that patients with cancer and no obesity (ie, BMI 20-25) have worse outcomes than patients with obesity.

**Objective:**

To assess the association between obesity and outcomes after a diagnosis of cancer.

**Data Sources:**

PubMed, the Cochrane Library, and EMBASE were searched from inception to January 2020.

**Study Selection:**

Studies reporting prognosis of patients with obesity using standard BMI categories and cancer were included. Studies that used nonstandard BMI categories, that were limited to children, or that were limited to patients with hematological malignant neoplasms were excluded. Screening was performed independently by multiple reviewers. Among 1892 retrieved studies, 203 (17%) met inclusion criteria for initial evaluation.

**Data Extraction and Synthesis:**

The Meta-analysis of Observational Studies in Epidemiology (MOOSE) and Preferred Reporting Items for Systematic Reviews and Meta-analyses (PRISMA) reporting guidelines were reporting guideline was followed. Data were extracted by multiple independent reviewers. Risk of death, cancer-specific mortality, and recurrence were pooled to provide an adjusted hazard ratio (HR) with a 95% CI . A random-effects model was used for the retrospective nature of studies.

**Main Outcomes and Measures:**

The primary outcome of the study was overall survival (OS) in patients with cancer, with and without obesity. Secondary end points were cancer-specific survival (CSS) and progression-free survival (PFS) or disease-free survival (DFS). The risk of events was reported as HRs with 95% CIs, with an HR greater than 1 associated with a worse outcome among patients with obesity vs those without.

**Results:**

A total of 203 studies with 6 320 365 participants evaluated the association of OS, CSS, and/or PFS or DFS with obesity in patients with cancer. Overall, obesity was associated with a reduced OS (HR, 1.14; 95% CI, 1.09-1.19; *P* < .001) and CSS (HR, 1.17; 95% CI, 1.12-1.23; *P* < .001). Patients were also at increased risk of recurrence (HR, 1.13; 95% CI, 1.07-1.19; *P* < .001). Conversely, patients with obesity and lung cancer, renal cell carcinoma, or melanoma had better survival outcomes compared with patients without obesity and the same cancer (lung: HR, 0.86; 95% CI, 0.76-0.98; *P* = .02; renal cell: HR, 0.74; 95% CI, 0.53-0.89; *P* = .02; melanoma: HR, 0.74; 95% CI, 0.57-0.96; *P* < .001).

**Conclusions and Relevance:**

In this study, obesity was associated with greater mortality overall in patients with cancer. However, patients with obesity and lung cancer, renal cell carcinoma, and melanoma had a lower risk of death than patients with the same cancers without obesity. Weight-reducing strategies may represent effective measures for reducing mortality in these patients.

## Key Points

**Question:**

Is obesity associated with better prognosis in patients with cancer?

**Findings:**

This meta-analysis of 203 studies with more than 6.3 million participants found that obesity was associated with increased overall and cancer-specific mortality, especially among patients with breast, colon, and uterine cancer. In contrast, patients with obesity and renal cell carcinoma, lung cancer, or melanoma had better survival than patients without obesity.

**Meaning:**

These findings suggest that survival outcomes are poor among patients with obesity and cancer, except in lung cancer and melanoma.

## Teaser

This systematic review and meta-analysis assesses the association between obesity and survival outcomes following a diagnosis of cancer.

## Introduction

Obesity, defined as a body mass index (BMI; calculated as weight in kilograms divided by height in meters squared) greater than 30, is a chronic disease with increasing prevalence around the world, largely contributing to important health issues in most countries.^1^ Alongside body fat, which is a general risk factor for serious illness (eg, metabolic syndrome), greater cardiometabolic risk has also been associated with the localization of excess fat in the visceral adipose tissue and ectopic deposits.^2^ Several large epidemiologic studies have evaluated the association between obesity and mortality. In particular, a meta-analysis of 230 cohort studies including more than 30 million individuals^3^ found that both obesity and overweight were associated with an increased risk of all-cause mortality. Despite the evidence that excess mortality increases with increasing BMI, some studies have reached the conclusion that elevated BMI may improve survival in patients with cardiovascular disease, a phenomenon called the obesity paradox.^4^

Increased BMI is also associated with an increased risk of multiple cancer types.^5^ In addition, obesity and overweight may increase cancer mortality.^6^ During last decades, we have observed a more rapid increase in obesity among adult cancer survivors compared with the general population.^7^ The mechanisms contributing to higher cancer incidence and mortality may include alterations in sex hormone metabolism, insulin and insulin-like growth factor levels, and adipokine pathways.^8,9^

Various studies have suggested that patients with cancer and a normal BMI (ie, 20-25) have worse outcomes than patients with obesity. This phenomenon (ie, the obesity paradox) in cancer is not well understood and presents controversial explanations.^10,11,12^ Three different meta-analyses have led to different results, in particular in lung and renal cell carcinomas.^13,14,15^ In lung cancer, obesity is favorably associated with long-term survival of surgical patients. Moreover, in renal cell carcinoma, an inconsistent association of BMI with cancer-specific survival (CSS) was found. Conversely, breast, ovarian, and colorectal cancer are invariably associated with increased mortality in patients with obesity.^16,17,18^ The main explanations for these observations include the general poor health status of patients with very low BMI. Additionally, weight loss may be associated with frailty and other risk factors (eg, smoking).^11^ In cancer, obesity is also associated with increased efficacy of programmed cell death 1 and programmed cell death ligand 1 (PD-1/PD-L1) blockade in both tumor-bearing mice and patients.^12^ This updated systematic review and meta-analysis was conducted to evaluate the prognosis of patients with cancer who have obesity vs those without obesity.

## Methods

### Search Strategy and Inclusion Criteria

We followed the Preferred Reporting Items for Systematic Reviews and Meta-analyses (PRISMA) reporting guideline and the Meta-analysis of Observational Studies in Epidemiology (MOOSE) reporting guideline^19,20^ A systematic search was conducted of EMBASE, PubMed, and the Cochrane Library for articles published from database inception until September 30, 2020. The following search terms were used: *((carcinoma or cancer or sarcoma or melanoma or (“Neoplasms”[MESH])) AND (obese OR obesity OR 30 kg/m^2^ OR “body mass index”) AND (hazard ratio) AND survival AND (multivariate OR cox or multivariable)*. The reference lists of identified articles were then manually searched to identify potentially relevant omitted citations. Articles that were not published in English were not included.

Retrospective and observational studies (ie, cohort, case-control) or prospective trials were selected when they reported the association of obesity, defined as a BMI of at least 30, with the risk of death (ie, overall survival [OS]), CSS, disease-free survival (DFS), or progression-free survival (PFS) in patients with cancer compared with counterparts without obesity (ie, BMI <30). We placed no restrictions on study setting, size, race, or country. Included studies were limited to those reporting hazard ratios (HRs) and their corresponding 95% CIs. Studies were restricted to adult patients with solid tumors. Hematologic malignant neoplasms were excluded. Short-term survival studies (eg, postsurgical mortality) were also excluded. Baseline-only BMI evaluation was considered (eg, BMI captured at cancer diagnosis in early-stage cancers or at metastatic disease in advanced-stage cancers).

The most up-to-date versions of full-text publications were included. Study selection was performed in 2 stages. First, titles and abstracts were screened; then, selected full-text articles were included according to the eligibility criteria. If pooled analyses of more than 1 study were evaluated for inclusion, the included articles were manually evaluated for duplicate inclusion compared with the other eligible articles. Screening was performed independently by 10 authors (M.G., G.T., A.G., A. Indini, A.C., O.N., V.R., A. Iaculli, L.D., M.S.), and conflicts were handled by consensus with a senior author (F.P.).

### Data Collection and Quality Assessment

Data were collected independently by using a predesigned spreadsheet (Excel version 2007 [Microsoft Corp]). Collected data items included authors, year of publication, study setting and design, median follow-up, treatments received, outcomes, and type of analysis. The primary outcome was OS; secondary end points were CSS and PFS or DFS. Along with data extraction, 1 author (F.P.) assessed study quality according to a modified Newcastle Ottawa Scale (NOS; range 1-9, with 1-3 indicating low quality, 4-6 indicating moderate quality, and 7-9 indicating high quality).^21^

### Statistical Analysis

First, pooled HRs with 95% CIs were estimated using random-effects meta-analysis with the generic inverse-variance method for studies that provided fully adjusted HRs. Inconsistency across studies was measured with the *I*^2^ method. Cutoff values of 25%, 50%, and 75% indicated low, moderate, and high heterogeneity, respectively. When *I*^2^ was larger than 50%, a random-effects model was primarily used because of the retrospective nature of included studies. To examine heterogeneity, we performed exploratory analyses of predefined subgroups based on type of disease, type of study, duration of follow-up, and race. Additionally, to address potential bias and verify our results, we performed sensitivity analyses using a leave-one-out method and the trim-and-fill method.^22^ These methods explore whether there are potential dominant studies that may have driven the results. Finally, to investigate the risk of publication bias, we applied the Egger test and visually inspected the funnel plots (ie, the Begg test).^23^ If the distribution of studies is symmetrical, the meta-analysis most likely does not have problems with publication bias. All statistical tests were 2-sided using a significance level of *P* < .05. All analyses were carried out using Comprehensive Meta-Analysis software version 3.3.070.

## Results

Our literature search yielded 1892 articles, of which 203 (17%) met the inclusion criteria for our overall systematic review of the association of obesity with cancer outcomes (Figure). Most excluded studies did not use the prespecified cutoff value for obesity (ie, BMI values different from 30 in 437 studies) or used a continuous cutoff for risk of death (eg, 1 unit-increase in BMI in 235 studies). Of the 203 articles, 170 (84%) were eligible for inclusion in the systematic review of the association of obesity with OS, 109 (54%) for association with CSS, and 79 (39%) for association with DFS or PFS. Descriptive data for studies included in our meta-analysis are listed in Table 1.^12,24,25,26,27,28,29,30,31,32,33,34,35,36,37,38,39,40,41,42,43,44,45,46,47,48,49,50,51,52,53,54,55,56,57,58,59,60,61,62,63,64,65,66,67,68,69,70,71,72,73,74,75,76,77,78,79,80,81,82,83,84,85,86,87,88,89,90,91,92,93,94,95,96,97,98,99,100,101,102,103,104,105,106,107,108,109,110,111,112,113,114,115,116,117,118,119,120,121,122,123,124,125,126,127,128,129,130,131,132,133,134,135,136,137,138,139,140,141,142,143,144,145,146,147,148,149,150,151,152,153,154,155,156,157,158,159,160,161,162,163,164,165,166,167,168,169,170,171,172,173,174,175,176,177,178,179,180,181,182,183,184,185,186,187,188,189,190,191,192,193,194,195,196,197,198,199,200,201,202,203,204,205,206,207,208,209,210,211,212,213,214,215,216,217,218,219,220,221,222,223,224,225^ Overall, the included studies included a total of 6 320 365 patients. Sample sizes ranged from 41 to 1 096 492 patients, with a median of 1543. Most studies were retrospective in nature (132 studies [63%]); the minority were prospective cohort or observational studies (63 studies [31%]) or pooled analyses or randomized trials (8 studies [4%]). Multivariable analysis was performed in 197 studies. Overall, 136 studies (63%) reported a significant association of obesity with the outcome in at least 1 end point. The mean NOS score was 7 (median, 7.5; range, 5-9), indicating that the overall quality of articles was good.

**Figure.**
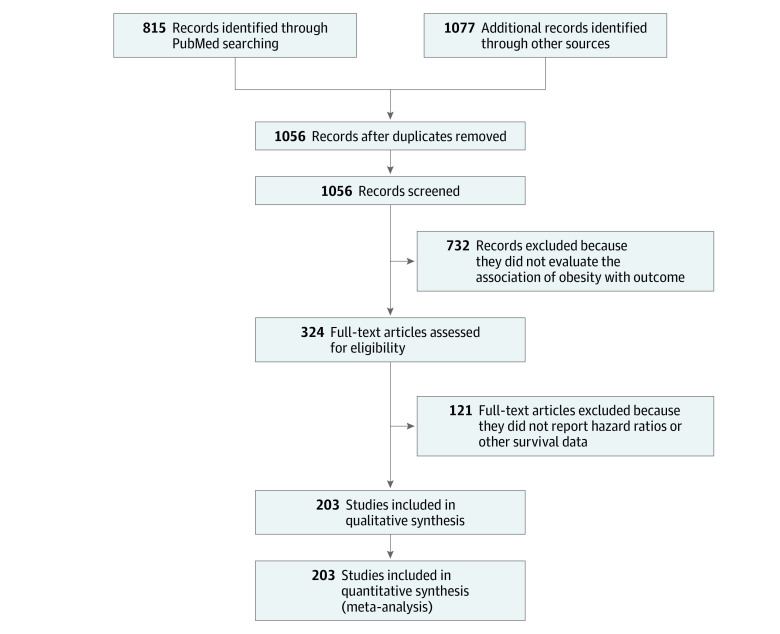
Flow Diagram of Included Studies

**Table 1.** Characteristics of Included Studies

Source	Patients, No.	Patients with obesity, No. (%)	Study type	Country	Disease	Follow-up, median, mo	Disease stage	Treatment	Type of analysis	OS	CSS	DFS/PFS	Quality^a^
Chromecki et al,^24^ 2013	4118	1739 (42)	Retrospective	Various	Bladder	44	Early	S with or without adjuvant CT	MVA	✓	✓	✓	7
Ferro et al,^25^ 2019	1155	224 (21)	Retrospective	Italy	Bladder	48	Early	TURBT with BCG vaccine	MVA		✓	✓	7
Siegel et al,^26^ 2013	853	216 (25)	Prospective	United States	Brain	19	Early	NA	MVA	✓			8
Abrahamson et al,^27^ 2006	1254	NA	Retrospective	United States	Breast	NA	Early and advanced	NA	MVA	✓			5
Abukabar et al,^28^ 2018	3012	433 (13)	Retrospective	Malaysia	Breast	24	Early and advanced	S with or withouth RT and/or CT	MVA	✓		✓	6
Alarfi et al,^29^ 2017	82	27 (33)	Prospective	Syria	Breast	40	Advanced	CT	MVA	✓		✓	6
Alsaker et al,^30^ 2011	2640	432 (16)	Retrospective	Norway	Breast	69	Early and advanced	NA	MVA		✓		7
Arce-Salinas et al,^31^ 2014	819	596 (74)	Retrospective	Mexico	Breast	28	Advanced	CT	UVA	✓		✓	6
Beasley et al,^32^ 2012	13 302	2330 (18)	Pooled analysis, meta-analysis	United States, China	Breast	NA	Early	All	MVA	✓	✓	✓	6
Blair et al,^33^ 2019	859	195 (23)	Cohort study	United States	Breast	94	Early and advanced	CT, RT, HT	MVA		✓		8
Braithwaite et al,^34^ 2010	2202	500 (23)	Retrospective	United States	Breast	88	Early	NA	MVA	✓	✓		8
Buono et al,^35^ 2017	841	231 (27)	Retrospective	Italy	Breast	58.9	Early	CT, HT, S	MVA	✓		✓	7
Caan and Kwan,^36^ 2008	1692	409 (24)	Retrospective	United States	Breast	NA	Early	S and/or adjuvant systematic therapy	MVA	✓	✓	✓	5
Cecchini et al,^37^ 2016	5265	1794 (34)	Phase 3, NSABP-B30	United States	Breast	108	Early	CT	MVA	✓			NA
Cecchini et al,^37^ 2016	2102	664 (32)	Phase 3, NSABP-B31	United States	Breast	99.6	Early	CT and TTZ	MVA	✓			8
Cecchini et al,^37^ 2016	3311	1186 (36)	Phase 3, NSABP-B34	United States	Breast	100.8	Early	BPS vs Placebo^b^	MVA	✓			8
Cecchini et al,^37^ 2016	4860	1917 (39)	Phase 3, NSABP-B38	United States	Breast	70.8	Early	CT	MVA	✓			7
Chang et al,^38^ 2000	177	64 (36)	Retrospective	United States	Breast	100	Advanced	Induction CT and S	MVA	✓			8
Chen et al,^39^ 2010	5042	283 (6)	Retrospective	China	Breast	6.5	Early and advanced	S, CT, RT, HT	MVA	✓	✓		6
Chung et al,^40^ 2017	8742	75 (9)	Retrospective	South Korea	Breast	92	Early	All	MVA	✓	✓		9
Cleveland et al,^41^ 2012	1447	319 (22)	Prospective, case-control	United States	Breast	NA	NA	NA	MVA	✓	✓		8
Connor et al,^42^ 2016	2478	142 (6)	Prospective, registry	United States	Breast	129.6	Early and advanced	NA	MVA	✓	✓		9
Conroy et al,^43^ 2011	3842	901 (23)	Prospective, cohort	United States	Breast	74.4	Early and advanced	S with or without CT/HT and/or RT	MVA	✓	✓		8
Copson et al,^44^ 2015	2843	533 (19)	Prospective	United Kingdom	Breast	70.4	Early and advanced	S, RT, CT, HT	MVA	✓		✓	8
Crozier et al,^45^ 2013	3017	1298 (43)	Retrospective	United States	Breast	63.6	Early	NA	MVA		✓	✓	8
Dal Maso et al,^46^ 2008	1453	172 (12)	Retrospective	Italy	Breast	NA	Early	S and/or adjuvant systematic therapy	UVA	✓	✓		5
Dignam et al,^47^ 2003	3385	395 (12)	Retrospective	United States	Breast	NA	Early	S and HT	MVA	✓	✓	✓	5
Dignam et al,^48^ 2006	4077	1056 (26)	Retrospective	United States	Breast	NA	NA	NA	MVA	✓	✓	✓	5
Elwood et al,^49^ 2018	1049	225 (21)	Retrospective	New Zealand	Breast	49.2	Early and advanced	CT, HT	MVA	✓	✓		6
Emaus et al,^50^ 2010	1364	147 (11)	Retrospective	Norway	Breast	98.4	Early and advanced	NA	MVA	✓	✓		8
Feliciano et al,^51^ 2017	1559	471 (30)	Retrospective	United States	Breast	108	Early	All	MVA		✓	✓	8
Goodwin et al,^52^ 2012	535	NA	Retrospective	Canada	Breast	145.2	Early	S with or without adjuvant CT and/or HT	MVA	✓		✓	9
He et al,^53^ 2012	1983	546 (28)	Retrospective	United States	Breast	47.6	Early and advanced	All	MVA	✓			7
Hellmann et al,^54^ 2010	528	76 (14)	Prospective	Denmark	Breast	93.6	Early and advanced	NA	MVA	✓	✓		7
His et al,^55^ 2016	3160	194 (6)	Prospective	France	Breast	109.2	Early	NA	MVA	✓	✓	✓	8
Jeon et al,^56^ 2015	41 021	1632 (4)	Prospective, registry	Korea	Breast	92	Early	CT, HT	MVA	✓	✓		8
Jiralerspong et al,^57^ 2013	6342	1779 (30)	Retrospective	United States	Breast	64.8	Early	NA	MVA	✓	✓	✓	7
Kawai et al,^58^ 2016	20 090	897 (5)	Prospective, registry	Japan	Breast	80.4	Early	CT, HT	MVA	✓	✓	✓	7
Keegan et al,^59^ 2010	4153	127 (3)	Retrospective	United States	Breast	NA	NA	NA	MVA	✓			5
Kwan et al,^60^ 2012	14 948	2440 (16)	Prospective, cohort	United States	Breast	93.6	Early	S with or without adjuvant CT and/or HT and/or RT	MVA	✓	✓		8
Kwan et al,^61^ 2014	11 351	3405 (30)	3 pooled case-control, 3 prospective cohort	United States	Breast	132	Early and advanced	All	MVA	✓	✓		8
Ladoire et al,^62^ 2014	5009	666 (13)	Pooled analysis, 2 phase 3	France	Breast	70.8	Early	CT	MVA	✓		✓	7
Larsen et al,^63^ 2015	1229	167 (14)	Prospective, cohort	Denmark	Breast	115.2	Early	NA	MVA	✓			9
Loi et al,^64^ 2005	1101	131 (12)	Retrospective	Australia	Breast	60	Early	S and/or adjuvant systematic therapy	MVA	✓		✓	6
Maskarinec et al,^65^ 2011	382	71 (19)	Prospective, cohort	Hawaii	Breast	158.4	Early and advanced	S with or without adjuvant CT and/or HT	MVA	✓	✓		9
McCullough et al,^66^ 2005	430 236	5433 (1)	Prospective	United States	Breast	240	Early and advanced	All	MVA		✓		9
McCullough et al,^67^ 2016	1308	301 (23)	Population-based	United States	Breast	180	Early	NA	MVA	✓	✓		9
Nichols et al,^68^ 2010	3993	639 (16)	Retrospective	United States	Breast	76.8	Early	S	MVA	✓	✓		7
Nur et al,^69^ 2019	49 259	202 (4)	Retrospective	Sweden	Breast	91.6	Early	S	MVA	✓	✓		8
Oh et al,^70^ 2011	747	251 (34)	Cohort study	Korea	Breast	62.2	Early	S with or without CT	MVA			✓	7
Oudanonh et al,^71^ 2020	3747	1790 (48)	Retrospective, registry	Canada	Breast	NA	Early	HT, CT, RT, anti-*ERBB2*	MVA	✓			5
Pajares et al,^72^ 2013	5683	1376 (24)	Retrospective	Spain	Breast	NA	Early	CT, HT, S	MVA	✓	✓	✓	5
Pfeiler et al,^73^ 2013	1509	315 (21)	Retrospective	Austria	Breast	60	Early	NA	MVA	✓	✓	✓	7
Pierce et al,^74^ 2007	1490	380 (26)	Prospective	United States	Breast	80.4	Early	S and/or adjuvant systematic therapy	MVA	✓			8
Probst-Hensch et al,^75^ 2010	855	72 (20)	Retrospective	Switzerland	Breast	43.8	Early	S with or without HT	MVA	✓			6
Senie et al,^76^ 1992	923	207 (22)	Prospective	United States	Breast	120	Early	NA	MVA			✓	9
Sparano et al,^77^ 2012	4817	1745 (46)	Retrospective, phase 3	United States	Breast	95	Early	S with CT and HT	MVA	✓	✓	✓	8
Sparano et al,^78^ 2012	6885	2547 (37)	Retrospective, 3 phase 3	United States	Breast	95	Early	S with or without CT and/or HT	MVA	✓	✓	✓	9
Su et al,^79^ 2013	1030	312 (30)	Randomized study	United States	Breast	NA	Early	S with or without CT and/or HT	MVA			✓	5
Sun et al,^80^ 2015	1109	410 (37)	Population-based	United States	Breast	162	Early	NA	MVA	✓	✓		8
Sun et al,^81^ 2018	1017	192 (19)	Retrospective	China	Breast	80	Early	CT, HT, RT, S	MVA	✓		✓	8
Tait et al,^82^ 2014	501	202 (40)	Retrospective	United States	Breast	40.1	Early and advanced	S, CT	MVA	✓		✓	6
Warren et al,^83^ 2016	878	NA	Retrospective	United States	Breast	129.6	Early	S	UVA			✓	9
Widschwendter et al,^84^ 2015	3754	788 (21)	Phase 3, SUCCESS A	Germany	Breast	NA	Early	CT with HT	MVA	✓		✓	5
Xiao et al,^85^ 2014	5785	1680 (29)	Retrospective	China	Breast	70	Early	CT, HT	MVA	✓			7
Mazzarella et al,^86^ 2013	1250	101 (8)	Retrospective	Europe	Breast, *ERBB2*	NA	Early	S, RT, CT	MVA	✓		✓	5
Rosenberg et al,^87^ 2009	2640	376 (14)	Retrospective	Sweden	Breast, HR positive	NA	Early and advanced	S, CT, HT, RT	MVA		✓		5
Ademuyiawa et al,^88^ 2011	418	164 (39)	Retrospective	United States	Breast, TN	37.2	Early	S	MVA	✓		✓	6
Dawood et al,^89^ 2012	2311	825 (36)	Retrospective	United States	Breast, TN	39	Early	S	MVA	✓	NA	✓	6
Melhem-Bertrandt et al,^90^ 2011	1413	460 (33)	Retrospective	United States	Breast, TN	59	Early	S with or without CT	MVA	✓	NA	✓	7
Frumovitz et al,^91^ 2014	3086	1026 (33)	Retrospective	United States	Cervical	133	Early and advanced	S, RT	MVA	✓	✓		9
Fedirko et al,^92^ 2014	3924	689 (18)	Prospective	Western Europe	CRC	49	Early and advanced	NA	MVA	✓	✓		7
Boyle et al,^93^ 2013	879	258 (29)	Retrospective	Australia	CRC	67.2	Early	NA	MVA	✓	✓	✓	7
Campbell et al,^94^ 2015	5615	1483 (26)	Prospective	Various	CRC	NA	Early and advanced	S, CT	MVA	✓	✓		5
Cespedes Feliciano et al,^95^ 2017	2470	NA	Retrospective	United States	CRC	72	Early	NA	MVA	✓	✓		7
Clark et al,^96^ 2013	99	40 (41)	Retrospective	United States	CRC	39.4	Early	CT and RT	MVA	✓		✓	6
Dahdaleh et al,^97^ 2018	1543	529 (34)	Retrospective, cohort	United States	CRC	30.9	Early	S, adjuvant CT	MVA	✓		✓	6
Dignam et al,^98^ 2006	4288	812 (10)	Retrospective	North America	CRC	NA	Early	CT	MVA	✓	✓	✓	5
Jayasekara et al,^99^ 2018	724	164 (23)	Cohort study	Australia	CRC	108	Early	NA	MVA	✓	✓		8
Kaidar-Person et al,^100^ 2015	184	46 (25)	Retrospective	Israel	CRC	27.6	Advanced	CT, bevacizumab	MVA	✓		✓	6
Kalb et al,^101^ 2019	612	127 (21)	Retrospective	Germany	CRC	58	Early	CT, RT, S	MVA	✓		✓	7
Meyerhardt et al,^102^ 2003	3561	500 (17)	Cohort study	United States	CRC	112	Early	S and CT	MVA	✓		✓	9
Meyerhardt et al,^103^ 2004	1688	306 (18)	Cohort study	United States	CRC	118	Early	S and CT	MVA	✓		✓	9
Meyerhardt et al,^104^ 2008	1043	236 (23)	Prospective	United States and Canada	CRC	63.6	Early	S and/or adjuvant systematic therapy	MVA	✓		✓	8
Morikawa et al,^105^ 2012	1060	200 (19)	Prospective, cohort	United States	CRC	162	Early and advanced	All	MVA	✓	✓		9
Ogino et al,^106^ 2009	546	84 (16)	Retrospective	United States	CRC	NA	Early and advanced	NA	MVA	✓	✓		5
Patel et al,^107^ 2015	1174	462 (39)	Retrospective	Australia	CRC	NA	Advanced	CT	MVA	✓			5
Pelser et al,^108^ 2014	5727	NA	Retrospective	United States	CRC	NA	Early and advanced	S, RT, CT	MVA	✓	✓		5
Prizment et al,^109^ 2010	1096	295 (27)	Retrospective, registry	United States	CRC	240	Early and advanced	CT, RT, S	MVA	✓	✓		9
Schlesinger et al,^110^ 2014	2143	397 (19)	Prospective	Germany	CRC	42	Early and advanced	NA	MVA	✓			6
Shah et al,^111^ 2015	242	59 (25)	Prospective	United States	CRC	NA	Advanced	S, CT	MVA	✓			7
Sinicrope et al,^112^ 2012	2693	630 (23)	Pooled analysis, randomized trial	United States	CRC	NA	Early	S with or without CT	MVA	✓		✓	5
Sinicrope et al,^113^ 2013	25 291	4463 (18)	Retrospective	United States	CRC	93.6	Early	NA	MVA	✓		✓	8
Sorbye et al,^114^ 2012	342	67 (20)	Retrospective	Europe	CRC	NA	Advanced	S with or without CT	MVA			✓	5
Wang et al,^115^ 2017	1452	NA	Retrospective	China	CRC	40.8	Early and advanced	All	MVA		✓		6
Zheng et al,^116^ 2016	226	52 (23)	Cohort study	China	CRC	NA	Early and advanced	NA	MVA	✓		✓	5
Doria-Rose et al,^117^ 2006	633	96 (15)	Retrospective	United States	CRC among women	112.8	Early	S and/or other, unspecified treatments	MVA	✓	✓		8
Kristensen et al,^118^ 2017	4330	NA	Retrospective	Denmark	Endometrial	NA	Early and advanced	S	MVA	✓			5
Nagle et al,^119^ 2018	1359	568 (42)	Retrospective	Australia	Endometrial	85.2	Early and advanced	S with or without CT	MVA	✓	✓		8
Nicholas et al,^120^ 2014	490	203 (41)	Retrospective	United States	Endometrial	54	Early and advanced	S	MVA	✓			6
Todo et al,^121^ 2014	716	99 (14)	Retrospective	Japan	Endometrial	74	Early and advanced	S, CT	MVA	✓	✓		7
Yoon et al,^122^ 2015	2987	417 (14)	Retrospective, cohort	United States	Endometrial	NA	Early and advanced	S, CT, RT	MVA	✓			5
Hynes et al,^123^ 2017	390	64 (17)	Prospective, cohort	Sweden	Esophagus	NA	Early	S	MVA	✓	✓		5
Spreafico et al,^124^ 2017	564	76 (13)	Retrospective	Canada	Esophagus	32.5	Early and advanced	All	MVA	✓		✓	6
Sundelöf et al,^125^ 2008	580	55 (10)	Retrospective	Sweden	Esophagus	NA	Early and advanced	NA	MVA	✓			5
Yoon et al,^126^ 2011	778	46 (19)	Retrospective	United States	Esophagus, adenocarcinoma	154.8	Early	S with or without adjuvant CT, RT and/or CTRT	MVA	✓	✓	✓	9
Thrift et al,^127^ 2012	783	263 (33)	Retrospective	Australia	Esophagus or gastric	76.8	Early and advanced	S with or without CTRT and/or BSC	MVA	✓			8
Trivers et al,^128^ 2005	1142	156 (4)	Retrospective	United States	Esophagus or gastric	NA	Early and advanced	S and/or other, unspecified treatments	MVA	✓		✓	5
Potharaju et al,^129^ 2018	392	40 (10)	Retrospective	India	GBM	48.6	NA	S, RT, and TMZ	MVA	✓			6
Gama et al,^130^ 2017	1279	243 (21)	Retrospective	Canada	HN	30	Early and advanced	RT, S, CT	MVA	✓		✓	6
Grossberg et al,^131^ 2016	190	65 (34)	Retrospective	United States	HN	68.6	Early	CT with RT	MVA	✓	✓	✓	7
Hu et al,^132^ 2019	576	33 (6)	Retrospective	China	HN, oral SCC	64	Early	S	MVA		✓	✓	7
Ata et al,^133^ 2019	8352	2841 (34)	Retrospective	United States	HCC	60	NA	Liver transplantation	MVA	✓	✓		7
Carr et al,^134^ 2018	521	NA	Retrospective	Italy	HCC	NA	Early and advanced	NA	MVA	✓			5
Yang et al,^135^ 2019	2442	86 (4)	Retrospective	United States	HCC	50.5	Early	S	MVA	✓		✓	8
Roque et al,^136^ 2016	128	72 (56)	Retrospective	United States	Leiomyosarcoma	49	Early and advanced	All	MVA	✓		✓	6
McMahon et al,^137^ 2017	1080	NA	Retrospective	United States	Liver	123.6	Early and advanced	All	MVA		✓		9
Abdel-Rahman,^138^ 2019	145 544	18 131 (24)	Population-based, randomized	United States	Lung	135	Early and advanced	NA	MVA	✓			9
Leung et al,^139^ 2011	58 931	3520 (6)	Prospective	Japan	Lung	NA	NA	NA	MVA		✓		7
Nonemaker et al,^140^ 2009	2054	Black participants: 50 (13); White participants: 46 (8)	Retrospective	United States	Lung	NA	NA	NA	MVA	✓	✓		5
Qi et al,^141^ 2009	420	79 (23)	Retrospective	United States	Lung	NA	Advanced	All	MVA	✓			5
Shepshelovich et al,^142^ 2019	29 217	418 (1)	Pooled analysis	Canada	Lung	NA	Early and advanced	NA	MVA	✓			5
Turner et al,^143^ 2011	188 699	22 054 (12)	Prospective	United States	Lung	312	NA	NA	MVA		✓		9
Xie et al,^144^ 2017	624	NA	Retrospective	China	Lung	63.2	Early	S	MVA	✓			6
McQuade et al,^145^ 2019	1918	513 (27)	Pooled analysis	United States	Melanoma	NA	Advanced	CT, IT, TT	MVA	✓		✓	5
Aldrich et al,^146^ 2013	501	126 (25)	Prospective (cohort)	United States	NSCLC	16	Early and advanced	NA	MVA	✓			7
Kichenadasse et al,^147^ 2020	1434	239 (7)	Prospective	Various	NSCLC	NA	Advanced	Atezolizumab vs docetaxel	UVA	✓		✓	7
Nakagawa et al,^148^ 2016	1311	25 (2)	Retrospective	Japan	NSCLC	59	Early	S	MVA	✓		✓	7
Bandera et al,^149^ 2015	1846	547 (30)	Cohort study	United States	Ovarian	NA	Early and advanced	CT	MVA	✓	✓		5
Kotsopoulos et al,^150^ 2012	1423	230 (18)	Retrospective	Canada	Ovarian	120	Early and advanced	All	MVA		✓		9
Minlikeeva et al,^151^ 2019	7022	1557 (22)	Retrospective, pooled data	United States and Australia	Ovarian	NA	Early and advanced	NA	MVA	✓		✓	5
Previs et al,^152^ 2014	81	28 (34)	Retrospective	United States	Ovarian	NA	Early and advanced	S, RT	MVA	✓	✓	✓	5
Tyler et al,^153^ 2012	425	28 (7)	Prospective, case-control	United States	Ovarian	116.4	Early and advanced	All	MVA	✓	✓		9
Yang et al,^154^ 2008	635	81 (13)	Prospective	Europe	Ovarian	96	Early and advanced	NA	MVA		✓		7
Dalal et al,^155^ 2012	41	8 (20)	Prospective	United States	Pancreas	NA	Advanced	CTRT	MVA	✓			7
Genkinger et al,^156^ 2015	1 096 492	NA	Cohort study	United States	Pancreas	152.4	NA	NA	MVA		✓		8
Gong et al,^157^ 2012	510	51 (10)	Retrospective	United States	Pancreas	121.2	Early and advanced	All	MVA	✓			9
Li et al,^158^ 2009	841	163 (19)	Retrospective	United States	Pancreas	NA	Early and advanced	NA	MVA	✓			5
Lin et al,^159^ 2013	799 542	19 988 (3)	Retrospective	Various	Pancreas	37.2	NA	NA	MVA		✓		6
Olson et al,^160^ 2010	475	108 (23)	Retrospective	United States	Pancreas	NA	Early and advanced	S	MVA	✓	✓		5
Yuan et al,^161^ 2013	902	136 (15)	Prospective, cohort	United States	Pancreas	480	Early and advanced	NA	MVA	✓			9
Tsai et al,^162^ 2010	795	103 (13)	Retrospective	United States	Pancreas	NA	Early and advanced	S	MVA	✓			5
Bassett et al,^163^ 2012	16 525	247 (18)	Prospective, cohort	Australia	Prostate	180	NA	NA	MVA		✓		9
Bonn et al,^164^ 2014	4376	483 (11)	Retrospective	Sweden	Prostate	48	Early	S, RT	MVA	✓	✓	✓	6
Dickerman et al,^165^ 2017	5158	564 (11)	Retrospective	United States	Prostate	NA	Early	All	MVA		✓		5
Efstathiou et al,^166^ 2007	945	145 (15)	Prospective	United States	Prostate	97.2	Advanced	RT with or without goserelin	MVA	✓	✓		8
Farris et al,^167^ 2018	987	192 (19)	Prospective, cohort	Canada	Prostate	228	Early and advanced	S, RT, HT	MVA	✓	✓		9
Froehner et al,^168^ 2014	2131	356 (17)	Retrospective	Germany	Prostate	110	Early	All	UVA, MVA	✓	✓		9
Gong et al,^169^ 2007	752	128 (17)	Retrospective	United States	Prostate	116.4	Early and advanced	S, ADT, RT, and other, unspecified treatments	MVA		✓		9
Han et al,^170^ 2010	2511	211 (8)	Retrospective	United States	Prostate	156	Early	All	UVA	✓			9
Ho et al,^171^ 2012	1038	337 (32)	Retrospective	United States	Prostate	41	Early and advanced	S	MVA			✓	6
Kelly et al,^172^ 2016	7822	1612 (21)	Retrospective	United States	Prostate	156	Early and advanced	All	MVA		✓		9
Kenfield et al,^173^ 2015	112 185	9984 (9)	Prospective	United States	Prostate	170	NA	NA	MVA		✓		9
Khan et al,^174^ 2017	822	NA	Retrospective	United States	Prostate	60	Early and advanced	All	MVA			✓	7
Ma et al,^175^ 2008	2546	87 (3)	Retrospective	United States	Prostate	84	Early and advanced	NA	MVA	✓	✓		7
Maj-Hes et al,^176^ 2017	6519	2462 (38)	Retrospective	Austria	Prostate	28	Early	S	MVA			✓	6
Møller et al,^177^ 2015	26 877	4140 (15)	Cohort study	Denmark	Prostate	43.2	Early and advanced	NA	MVA		✓		8
Rudman et al,^178^ 2016	273	59 (22)	Retrospective	United Kingdom	Prostate	139.2	Early	HT	MVA	✓	✓		9
Schiffmann et al,^179^ 2017	16 014	2403 (15)	Retrospective	Germany	Prostate	36.4	Early	S	MVA			✓	6
Spangler et al,^180^ 2007	924	286 (31)	Prospective	Various	Prostate	36	Early	S	MVA			✓	7
Vidal et al,^181^ 2017	4268	1372 (32)	Retrospective	United States	Prostate	81.6	Early	S	MVA		✓	✓	8
Wu et al,^182^ 2015	333	118 (35)	Retrospective	United States	Prostate	NA	Advanced	CT	MVA	✓			5
Montgomery et al,^183^ 2007	1006	160 (16)	Retrospective	United States	Prostate, AD	NA	Advanced	Bilateral orchiectomy with or without flutamide	MVA	✓		✓	5
Halabi et al,^184^ 2007	1296	405 (31)	Retrospective	United States	Prostate, AI	33.8	Early and advanced	NA	MVA	✓	✓		6
Montgomery et al,^183^ 2007	671	253 (38)	Retrospective	United States	Prostate, AI	NA	Advanced	Mitoxantrone and prednisone vs docetaxel and estramustine	MVA	✓		✓	5
Keizman et al,^185^ 2014	278	67 (24)	Retrospective	Israel	RCC	55	Advanced	TKI	MVA	✓		✓	7
Lee et al,^186^ 2010	2750	120 (4)	Retrospective	South Korea	RCC	34.8	Early	S	MVA		✓	✓	6
Parker et al,^187^ 2006	970	336 (35)	Retrospective	United States	RCC	56.4	Early	S	MVA	✓	✓		7
Psutka et al,^188^ 2016	387	166 (43)	Retrospective	United States	RCC	86.4	Early	S	MVA	✓	✓	✓	8
Spiess et al,^189^ 2012	99	43 (43)	Retrospective	United States	RCC	44.4	Early and advanced	S	MVA	✓			6
Yu et al,^190^ 1991	360	44 (12)	Retrospective	United States	RCC	53	Early	S	MVA	✓			7
Hung et al,^191^ 2018	33 551	2362 (7)	Retrospective, cohort	Taiwan	Solid cancers	43.8	Early and advanced	S	MVA	✓	✓		6
Houdek et al,^192^ 2019	261	71 (9)	Retrospective	Canada	STS	48	NA	RT vs none	MVA	✓		✓	6
Iyengar et al,^193^ 2014	155	30 (19)	Retrospective	United States	Tongue	NA	Early	S	MVA	✓	✓	✓	5
Xu et al,^194^ 2019	644	92 (14)	Retrospective	China	Upper tract urothelial	39	Early	S	MVA	✓	✓	✓	6
Arem et al,^195^ 2013	1400	610 (43)	Retrospective	United States	Uterine	61.2	Early and advanced	NA	MVA	✓	✓		7
Matsuo et al,^196^ 2016	665	459 (69)	Retrospective	United States	Uterine	36.4	Early and advanced	S with CTRT	MVA	✓		✓	6
Ruterbusch et al,^197^ 2014	627	184 (29)	Retrospective	United States	Uterine	NA	Early and advanced	S with or without CT	MVA	✓	✓		5
Seidelin et al,^198^ 2016	3638	984 (27)	Population-based	Denmark	Uterine	NA	Early and advanced	NA	MVA	✓			7
Abdullah et al,^199^ 2011	5036	567 (11)	Retrospective, cohort	Various	Various	NA	NA	NA	MVA		✓		5
Akinyemiju et al,^200^ 2018	22 514	8786 (39)	Prospective	United States	Various	78	NA	NA	MVA		✓		8
Barroso et al,^201^ 2018	54 446	15 158 (28)	Retrospective	Spain	Various	NA	NA	NA	MVA	✓	✓		5
Boggs et al,^202^ 2011	51 695	23 656 (46)	Prospective	United States	Various	NA	NA	NA	MVA		✓		8
Cortellini et al,^203^ 2019	976	377 (39)	Retrospective	Italy	Various	17.2	Advanced	Anti–PD-1/PD-L1	MVA	✓		✓	6
Drake et al,^204^ 2017	7061	3220 (46)	Prospective, cohort	Sweden	Various	202	Early and advanced	All	MVA	✓	✓		9
Han et al,^205^ 2014	13 901	708 (5)	Retrospective	United States	Various	NA	NA	NA	MVA		✓		5
Izumida et al,^206^ 2019	10 824	235 (2)	Cohort study	China	Various	220.8	NA	NA	MVA		✓		9
Janssen et al,^207^ 2015	927	NA	Retrospective	United States	Various	NA	Early and advanced	NA	MVA	✓			5
Jenkins et al,^208^ 2018	502 631	12 539 (25)	Cohort study	United Kingdom	Various	93.6	NA	NA	MVA	✓			7
Katzmarzyk et al,^209^ 2012	10 522	1972 (19)	Retrospective	Canada	Various	168	Early and advanced	All	MVA		✓		8
Kitahara et al,^210^ 2014	313 575	9564 (3)	Retrospective	Various	Various	NA	NA	NA	MVA		✓		5
Martini et al,^211^ 2020	90	23 (26)	Retrospective	United States	Various	NA	Advanced	IT	MVA	✓		✓	5
Mathur et al,^212^ 2010	279	97 (35)	Retrospective	United States	Various	31	Advanced	Hepatectomy	MVA	✓		✓	6
Meyer et al,^213^ 2015	35 703	2820 (8)	Population-based	Switzerland	Various	NA	Early and advanced	NA	MVA	✓			7
Nechuta et al,^214^ 2010	71 243	8264 (12)	Cohort study	China	Various	109.2	NA	NA	MVA		✓		9
Parr et al,^215^ 2010	401 215	16 978 (4)	Retrospective	All	Various	NA	NA	NA	MVA	✓			5
Sasazuki et al,^216^ 2011	353 422	7327 (2)	Prospective, cohort	Japan	Various	150	NA	NA	MVA		✓		9
Silventoinen et al,^217^ 2014	734 438	9187 (1)	Retrospective	Finland, Sweden	Various	403.2	NA	NA	MVA	✓	✓		9
Song et al,^218^ 2012	135 745	NA	Prospective	Europe	Various	201.6	NA	NA	MVA		✓		9
Taghizadeh et al,^219^ 2015	8645	683 (8)	Cohort study	Netherlands	Various	480	NA	NA	MVA	✓			9
Tseng,^220^ 2013	89 056	NA	Prospective	Taiwan	Various	144	Early and advanced	All	MVA		✓		9
Tseng,^221^ 2016	92 546	NA	Retrospective	Taiwan	Various	204	Early and advanced	All	MVA		✓		9
Valentijn et al,^222^ 2013	10 247	1851 (18)	Retrospective	The Netherlands	Various	64.8	NA	NA	MVA		✓		7
Wang et al,^12^ 2019	250	81 (12)	Retrospective	United States	Various	NA	Advanced	IT	MVA	✓		✓	5
Xu et al,^223^ 2018	6197	1885 (30)	Cohort study	United States	Various	204	NA	NA	MVA		✓		9
Yano et al,^224^ 2013	3641	792 (22)	Prospective	Japan	Various	122	NA	NA	MVA		✓		9
You et al,^225^ 2015	1314	NA	Prospective, cohort	China	Various	52.7	Early and advanced	NA	MVA	✓		✓	7

Abbreviations: AD, androgen dependent; ADT, androgen deprivation therapy; AI, androgen independent; BCG, Bacillus Calmette-Guérin; BPS, bisphosphonate; BSC, best supportive care; CRC, colorectal cancer; CSS, cancer specific survival; CT, chemotherapy; CTRT, chemotherapy with radiotherapy; DFS, disease-free survival; GBM, glioblastoma multiforme; HCC, hepatocellular carcinoma; HN, head and neck tumors; HR, hormone receptor; HT, hormone therapy; IT, immunotherapy; NSCLC, non–small cell lung cancer; MVA, multivariate analysis; NA, not applicable; OS, overall survival; PD-1, programmed cell death 1; PD-L1, programmed cell death ligand 1; PFS, progression-free survival; RCC, renal cell carcinoma; RT, radiotherapy; STS, soft tissue sarcoma; SCC, squamous cell carcinoma; S, surgery; TKI, tyrosine kinase inhibitor; TMZ, temozolomide; TN, triple negative; TT, targeted therapy; TTZ, trastuzumab; TURBT, transurethral resection of bladder tumor; UVA, univariate analysis.

^a^ Quality assessed according to a modified Newcastle Ottawa Scale (range 1-9, with 1-3 indicating low quality, 4-6 indicating moderate quality, and 7-9 indicating high quality).

^b^ Clinical trial randomizing breast cancer patients to receive BPS vs placebo; patients received concomitant systemic anticancer treatment according to physicians’ decision (following institutional guidelines).

### OS and Obesity in Patients With Cancer

A total of 170 studies reported data on OS. Because the heterogeneity test showed a high level of heterogeneity (*I*^2^ = 79.7%; *P* < .001) among studies, a random-effects model was used for the analysis. OS among patients with obesity was significantly worse than that among patients without obesity (HR, 1.14; 95% CI, 1.09-1.19; *P* < .001) (eFigure 1 in the Supplement). The association of obesity with outcomes was independent by other main cancer prognostic factors, including stage (100%), sex (85%), age (100%), race (80%), smoking status (83%), and other comorbidities according to multivariable analysis.

### CSS and Obesity in Patients With Cancer

Similarly, obesity was associated with reduced CSS in 109 studies (HR, 1.17; 95% CI, 1.12-1.23; *P* < .001) (eFigure 2 in the Supplement). Heterogeneity was high (*I*^2^ = 73.9%; *P* < .001), so a random-effects model was used.

### DFS or PFS and Obesity in Patients With Cancer

In 79 studies, obesity was associated with worse DFS or PFS compared with not having obesity (HR, 1.13; 95% CI, 1.07-1.19; *P* < .001) (eFigure 3 in the Supplement). Heterogeneity was high (*I*^2^ = 73.7%; *P* < .001), so a random-effects model was used.

### Subgroup Analysis

A subgroup analysis for OS was performed according to type of disease (Table 2, Table 3, and Table 4). Patients with breast, colorectal, or uterine cancers and obesity had higher overall mortality than those without obesity (breast: HR, 1.26; 95% CI, 1.2-1.33; *P* < .001; colorectal: HR, 1.22; 95% CI, 1.14-1.31; *P* < .001; HR, 1.20; 95% CI, 1.04-1.38; *P* = .01). Patients with obesity and lung cancer, renal cell carcinoma, or melanoma had better survival outcomes compared with patients without obesity and the same cancer (lung: HR, 0.86; 95% CI, 0.76-0.98; *P* = .02; renal cell: HR, 0.74; 95% CI, 0.53-0.89; *P* = .02; melanoma: HR, 0.74; 95% CI, 0.57-0.96; *P* < .001). CSS was decreased in patients with obesity and breast, colorectal, prostate, and pancreatic cancers (breast: 1.23; 95% CI, 1.15-1.32; *P* < .001; colorectal: HR, 1.24; 95% CI, 1.16-1.32; *P* < .001; prostate: HR, 1.26; 95% CI, 1.08-1.47; *P* = .01; pancreatic: HR, 1.28; 95% CI, 1.05-1.57; *P* = .01). DFS was decreased in patients with obesity and breast, colorectal, prostate, and gastroesophageal cancers (breast: HR, 1.14; 95% CI, 1.1-1.19; *P* < .001; colorectal: HR, 1.15; 95% CI, 1.01-1.3; *P* = .01; prostate: HR, 1.29; 95% CI, 1.07-1.56; *P* < .001; gastroesophageal: HR, 1.62; 95% CI, 1.13-2.32; *P* < .001). Additional subgroup analyses included type of study (retrospective: HR, 1.07; 95% CI, 1.07-1.18; *P* < .001; prospective: HR, 1.14; 95% CI, 1.05-1.23; *P* < .001), duration of follow up (>10 years: HR, 1.16; 95% CI, 0.86-1.58; *P* = .08; <10 years: HR, 1.23; 95% CI, 0.84-1.63; *P* = .09), race (non-Asian race: HR, 1.22; 95% CI, 0.86-1.66, *P* = .06; Asian race: HR, 1.22; 95% CI, 0.74-1.72; *P* = .09), and stage of disease (early: HR, 1.20; 95% CI, 0.99-1.25; *P* = .07; advanced: HR, 1.2; 95% CI, 1.12-1.28; *P* = .01). Regression analysis according to NOS score was not significant.

**Table 2.** Association of Obesity With Overall Mortality, by Cancer

Disease	Studies, No.	HR (95% CI)	*P* value	*I*^2^ %	Type of analysis
Bladder or UTUC	3	1.08 (0.98-1.20)	.11	0	Random
Brain	2	0.96 (0.50-1.84)	.90	88.5	Random
Breast	59	1.26 (1.20-1.33)	.004	51.3	Random
CRC	30	1.22 (1.14-1.31)	.001	54.5	Random
Gastroesophageal	7	1.08 (0.77-1.52)	.62	80.2	Random
Head and neck	7	0.59 (0.33-1.05)	.07	65.4	Random
Hepatobiliary	5	1.06 (0.89-1.25)	.48	73.6	Random
Lung	11	0.86 (0.76-0.98)	.02	60.4	Random
Melanoma	1	0.74 (0.63-0.89)	.004	0	Random
Ovarian	4	1.03 (0.75-1.41)	.84	64.7	Random
Pancreas	6	1.36 (0.95-1.93)	.08	80.5	Random
Prostate	12	1.07 (0.91-1.25)	.38	69.7	Random
RCC	5	0.78 (0.57-0.96)	.02	89.5	Random
Uterine	12	1.20 (1.04-1.38)	.01	60.8	Random
Various	9	1.10 (1.05-1.16)	.008	96.1	Random

Abbreviations: CRC, colorectal cancer; HR, hazard ratio; RCC, renal cell carcinoma; UTUC, upper tract urothelial carcinoma.

**Table 3.** Association of Obesity With Cancer-Specific Mortality by Cancer Type

Disease	Studies, No.	HR (95% CI)	*P* value	*I*^2^, %	Type of analysis
Bladder or UTUC	3	1.36 (0.96-1.93)	.08	59.4	Random
Breast	36	1.23 (1.15-1.32)	.004	58.8	Random
CRC	13	1.24 (1.16-1.33)	.002	0	Random
Gastroesophageal	2	0.83 (0.58-1.16)	.28	0	Random
Head and neck	3	1.35 (0.27-6.74)	.70	90.5	Random
Hepatobiliary	1	0.79 (0.50-1.24)	.31	0	Random
Lung	3	0.53 (0.30-0.92)	.02	0	Random
Ovarian	4	1.06 (0.82-1.37)	.61	33.3	Random
Pancreas	3	1.28 (1.05-1.57)	.01	61.1	Random
Prostate	15	1.26 (1.08-1.47)	.001	57.9	Random
RCC	4	1.08 (0.58-2.00)	.80	89.5	Random
Uterine	6	1.02 (0.75-1.39)	.86	69.1	Random
Various	16	1.08 (0.97-1.19)	.14	83.3	Random

Abbreviations: CRC, colorectal cancer; HR, hazard ratio; RCC, renal cell carcinoma; UTUC, upper tract urothelial carcinoma.

**Table 4.** Association of Obesity With Recurrence by Cancer Type

Disease	Studies, No.	HR (95% CI)	*P* value	*I*^2^, %	Type of analysis
Bladder or UTUC	3	1.42 (0.92-2.20)	.11	88.3	Random
Breast	34	1.14 (1.10-1.19)	.002	0	Random
CRC	12	1.15 (1.01-1.30)	.02	67.6	Random
Gastroesophageal	1	1.62 (1.13-2.32)	.005	0	Random
Head and neck	3	1.03 (0.48-2.20)	.92	75.7	Random
Hepatobiliary	2	1.06 (0.73-1.53)	.73	88.9	Random
Lung	2	0.55 (0.18-1.62)	.28	77.5	Rando
Melanoma	1	0.79 (0.69-0.90)	.006	0	Random
Ovarian	2	1.04 (0.92-1.17)	.52	0	Random
Prostate	11	1.29 (1.07-1.56)	.003	85.1	Random
RCC	4	0.69 (0.41-1.14)	.15	62.4	Random
Sarcoma	1	0.89 (0.47-1.68)	.72	0	Random
Uterine	2	0.98 (0.45-2.11)	.97	74.3	Random
Various	1	0.72 (0.49-1.05)	.09	0	Random

Abbreviations: CRC, colorectal cancer; HR, hazard ratio; RCC, renal cell carcinoma; UTUC, upper tract urothelial carcinoma.

### Publication Bias

A funnel plot was used to assess publication bias in the studies evaluating OS in patients with and without obesity. No publication bias was detected by funnel plot inspection (Begg test). Egger test was instead significant (eFigure 4 in the Supplement). According to the trim-and-fill method, 18 studies were placed to the left of the mean, and according the random-effect model, the final result for OS was similar (HR, 1.08; 95% CI, 1.03-1.13). After the leave-one-out procedure, HRs for OS ranged from 1.14 to 1.15.

## Discussion

This meta-analysis found that overall mortality was increased in patients with obesity and breast, colorectal, or uterine cancers. Cancer mortality was increased in breast, colorectal, prostate, and pancreatic cancers. Finally, the relapse rate was increased in breast, colorectal, prostate and gastroesophageal cancers. The obesity paradox, which describes improved cancer and all-cause mortality rates among patients with obesity, was observed in lung cancer and in melanoma; however, these data derive from only 12 studies. We used a categorical BMI definition of obesity (ie, BMI ≥30), because a more standardized definition would permit the comparison and synthesis of studies better than other categories (eg, continuous measures or unit of BMI increase).

The magnitude of effect size was similar for both OS and CSS in breast, colorectal, and lung cancer. This means that obesity may affect both the natural history of cancer and noncancer-related deaths.

Various factors are potentially associated with increased cancer mortality in some malignant neoplasms. Hormonal factors, reduced physical activity, more lethal or aggressive disease behavior, metabolic syndromes, and potential undertreatment in patients with obesity are possible reasons. It is well known that postmenopausal women with higher BMI have an increased risk of breast cancer because of higher estrogen levels resulting from the peripheral conversion of estrogen precursors (from adipose tissue) to estrogen.^226^ In these patients, weight loss and exercise may reduce cancer risk by lowering exposure to breast cancer biomarkers.^227^ In colorectal cancer, prediagnosis BMI was associated with increased all-cause, cardiovascular, and colorectal cancer–specific mortality.^228^ The reason for this association is not presently understood, although insulin, insulin-like growth factors, their binding proteins, chronic inflammation, oxidative stress, and impaired immune surveillance have been supposed to be causative factors.^229^ In pancreatic cancer, higher prediagnostic BMI was associated with more advanced stage at diagnosis, with 72.5% of patients with obesity presenting with metastatic disease vs 59.4% of patients with reference-range BMI (*P* = .02) in 2 large prospective cohort studies.^161^ Lastly, in prostate cancer, obesity may be a consequence of androgen deprivation therapy but seems also associated with more aggressive disease (ie, Gleason score ≥7)^230^ or more advanced disease at diagnosis.^231^

Our results showed that patients with obesity and lung cancer had significantly prolonged CSS and OS compared with patients without obesity. When considering these findings, we must take into account that 9 of 11 evaluated studies included patients with advanced and/or metastatic disease. Cancer cachexia mechanisms are not completely defined, but research has shown that the systemic inflammatory status induced either by the tumor or host response is a key moment in the development of cachexia.^232^ Lung cancers are indeed known to be aggressive, and patients with advanced disease usually have poorer performance statuses and experience significant weight loss at the time of diagnosis, which underlies a systemic inflammatory response.^233^ In our studies, obesity was positively associated with OS, independent of smoking status, in patients with lung cancer. Interestingly, a post hoc pooled analysis of randomized prospective trials comparing a PD-L1 checkpoint inhibitor (atezolizumab) with docetaxel in patients with advanced non–small cell lung cancer (NSCLC), revealed that the OS benefit for patients with obesity vs those with reference-range BMI was restricted to patients who received immunotherapy; no association was found in the group receiving docetaxel.^147^ Another study also explored the role of baseline BMI and BMI variation during treatment in a cohort of patients with advanced NSCLC and PD-L1 expression of at least 50% who received first-line pembrolizumab (a PD-1 checkpoint inhibitor) and in a control cohort of patients with NSCLC receiving first-line standard chemotherapy, confirming that the survival benefit for patients with obesity was restricted to those receiving immunotherapy.^234^

Similar findings have been described in patients with melanoma receiving immunotherapy, and a survival benefit for patients with obesity was reported in the single study^205^ included in our meta-analysis. However, despite some evidence showing that patients with obesity and melanoma who were receiving immune-checkpoint inhibitors achieved better outcomes,^235,236^ the association is currently questioned, given that opposite results have been reported in a multicenter study.^237^

Interestingly, patients with obesity and renal cell carcinoma also had a significantly longer OS compared with the patients without obesity. It has been hypothesized that the perinephric white adipose tissue acts as a reservoir of activated immune cells, with increased characteristics of hypoxia, infiltration of T helper type 1 cells, regulatory T cells, dendritic cells, and type 1 macrophages. However, only 1 of 6 studies included patients who were receiving immunotherapy.^238,239^

Intriguingly, we found that the association between obesity and better clinical outcomes was confirmed for those malignant neoplasms in which immune checkpoint inhibitors have first (and strongly) proved to be effective; however, studies involving patients receiving immune checkpoint inhibitors are poorly represented in this meta-analysis. Such results might be an epiphenomenon; however, we speculate that white adipose tissue could be considered an immune organ, which somehow plays a role in the antitumor immune response. It has been observed that the adipocyte-derived hormone leptin could alter T cell function, resulting in improved response to anti–PD-1 therapy.^12^ Moreover, another preclinical study reported that white adipose tissue acts as a reservoir for a peculiar population of memory T cells, which elicit some effective responses in the case of antigenic re-exposure during infections (and why not in case of exposure to cancer-specific antigens?).^240^ Finally, considering that immune checkpoint inhibitors exert their action within the tumor microenvironment, modulating the interactions between the tumor and the host, it has been proposed that systemic metabolic conditions, including high blood cholesterol, obesity, hyperglycemia and diabetes, atherosclerosis, and hypertension, may represent the epiphenomena of an inflamed patient. Such a patient might be characterized by an enrichment of cytokines and pro-inflammatory mediators (both in the innate and adaptive compartments) and by a condition of T cell exhaustion, with defective cellular-mediated mechanisms. Nevertheless, in these patients, immune checkpoint blockade might be more effective in reversing this immunological anergy both at the tumor and at the systemic levels.^241^

Patients with obesity are also at increased risk of reduced physical activity. Various studies highlighted this concept. Physical activity decreases over time in patients with obesity.^242,243^ In particular, physical activity is strictly associated with breast cancer and colorectal cancer mortality.^244,245^ Therefore physical activity (or inactivity) should be a major target of obesity prevention and treatment in particular for patients with cancer. Type 2 diabetes is strongly associated with obesity in the metabolic syndrome. More than 80% of cases of type 2 diabetes can be attributed to obesity, which may also account for many diabetes-related deaths. The association between BMI and cause-specific mortality was also illustrated in the Prospective Studies Collaboration analysis.^246^ In the upper BMI range (ie, 25 to 50), each 5-unit increase in BMI was associated with a significant increase in mortality from coronary heart disease, stroke, diabetes, chronic kidney disease, and many cancers. In the same analysis, individuals with BMI less than 22.5 had higher mortality compared with individuals with a BMI of 22.5 to 25. The excess mortality was predominantly associated with smoking-related diseases (ie, respiratory disease and cancer). However, there are no clear recommendations about dosing of chemotherapy in patients with obesity, so caution is recommended for high-risk regimens.^247^ The hypothesis that a reduced dose according to ideal body weight may lead to a worse outcome cannot be confirmed by prospective studies but may be considered a potential reason for the observed results in some settings (eg, breast cancer). In a pooled analysis of toxic effects in patients with and without obesity, rates of toxic effects were similar or lower in patients with obesity.^248^

### Limitations

This study has several limitations. First, we combined data for patients with obesity and compared their prognosis with patients with different weights (ie, normal weight or normal weight and overweight). Second, accurate measures of potentially self-reported weight and height are always a challenge in observational studies. The evaluation often takes place before diagnosis, but in some studies the timing of the obesity diagnosis was not described. Patients with obesity have a generally poor prognosis in terms of overall mortality and noncancer mortality, so it seems obvious that their prognosis would be worse than patients without obesity. However, almost all studies provided a multivariate analysis according to main prognostic factor for oncological outcome so that obesity remains generally an independent prognostic factor in patients with cancer. The outcome was almost never adjusted for private medical insurance, but obesity can increase costs for cancer treatment and complications. Therefore, patients with a lower socioeconomic status may have had reduced access to medical facilities (ie, access to anticancer treatments), rehabilitation, or follow-up intensity and therefore had inferior outcomes. Duration of follow-up, treatments received, and countries were heterogeneous even if subgroup analyses did not explain results with these different variables. Furthermore, this meta-analysis compared mortality between patients belonging to a fixed category of obesity (ie, BMI >30), and thus, we are not able to provide an effect size per unit increment.

## Conclusions

In this study, the results supported the notion that obesity is a competing risk factor for overall and cancer specific mortality as well as recurrence in various cancers treated with curative intent or for metastatic disease, except for lung cancer and melanoma, in which obesity was associated with reduced mortality (obesity paradox). These results suggest that oncologists should increase their efforts to manage patients in multidisciplinary teams for care and cure of both cancer and obesity. Improving lifestyle factors (eg, physical activity, caloric intake, care and prevention of cardiovascular complications), more intensive follow-ups of cancer in patients with obesity, and adequate dose of medical therapies are all proven measures that may improve prognosis for patients with cancer and obesity.

## Notes

Supplement. **eFigure 1.** Forest Plot for Overall Survival **eFigure 2.** Forest Plot for Cancer-Specific Survival **eFigure 3.** Forest Plot for Disease-Free Survival **eFigure 4.** Funnel Plot for Overall Survival Analysis
