# Supplementary material for: Association of Obesity With Survival Outcomes in Patients With Cancer: A Systematic Review and Meta-analysis
Source: JAMA Netw Open. 2021 Mar 29;4(3):e213520. doi: 10.1001/jamanetworkopen.2021.3520 (PMC8008284; doi:10.1001/jamanetworkopen.2021.3520)
Supplement: Supplement. — eFigure 1. Forest Plot for Overall Survival eFigure 2. Forest Plot for Cancer-Specific Survival eFigure 3. Forest Plot for Disease-Free Survival eFigure 4. Funnel Plot for Overall Survival Analysis [file jamanetwopen-e213520-s001.pdf]

# Supplemental Online Content

Petrelli F, Cortellini A, Indini A, et al. Association of obesity with survival outcomes in patients with cancer: a systematic review and meta-analysis. *JAMA Netw Open*. 2021;4(3):e213520. doi:10.1001/jamanetworkopen.2021.3520

**eFigure 1.** Forest Plot for Overall Survival

**eFigure 2.** Forest Plot for Cancer-Specific Survival

**eFigure 3.** Forest Plot for Disease-Free Survival

**eFigure 4.** Funnel Plot for Overall Survival Analysis

This supplemental material has been provided by the authors to give readers additional information about their work.

© 2021 Petrelli F et al. *JAMA Network Open.*

## **eFigure 1.** Forest Plot for Overall Survival

| Study name | Subgroup within study | Outcome | Hazard ratio | Lower limit | Upper limit | Z-Value | p-Value |
|---|---|---|---|---|---|---|---|
| | | | Statistics for each study | | | | |
| Chromecki 2012 | Bladder/UTUC | os | 1,080 | 0,970 | 1,202 | 1,404 | 0,160 |
| Meyer 2015 | Bladder/UTUC | os | 1,160 | 0,620 | 2,170 | 0,464 | 0,642 |
| Xu 2018 | Bladder/UTUC | os | 1,110 | 0,780 | 1,580 | 0,580 | 0,562 |
| Potharaju 2018 | Brain | os | 0,680 | 0,470 | 0,984 | -2,046 | 0,041 |
| Siegel 2013 | Brain | os | 1,320 | 1,040 | 1,675 | 2,282 | 0,022 |
| Abrahamson 2006 | Breast | os | 1,480 | 1,090 | 2,010 | 2,512 | 0,012 |
| Abubakar 2018 | Breast | os | 1,180 | 0,810 | 1,719 | 0,862 | 0,389 |
| Ademuyiwa 2011 | Breast | os | 0,940 | 0,540 | 1,636 | -0,219 | 0,827 |
| Alarfi 2017 | Breast | os | 0,540 | 0,260 | 1,122 | -1,652 | 0,098 |
| Arce-Salinas 2014 | Breast | os | 1,790 | 1,150 | 2,786 | 2,579 | 0,010 |
| Beasley 2012 | Breast | os | 0,860 | 0,700 | 1,057 | -1,436 | 0,151 |
| Braithwaite 2010 | Breast | os | 1,290 | 0,700 | 2,377 | 0,816 | 0,414 |
| Buono 2017 | Breast | os | 1,250 | 0,330 | 4,735 | 0,328 | 0,743 |
| Caan 2008 | Breast | os | 1,300 | 0,800 | 2,113 | 1,059 | 0,290 |
| Cecchini 2016 | Breast | os | 1,080 | 0,990 | 1,178 | 1,734 | 0,083 |
| Chang 2000 | Breast | os | 1,340 | 0,880 | 2,040 | 1,364 | 0,173 |
| Chen 2010 | Breast | os | 1,550 | 1,100 | 2,184 | 2,505 | 0,012 |
| Chung 2017 | Breast | os | 1,270 | 0,900 | 1,792 | 1,360 | 0,174 |
| Cleveland 2011 | Breast | os | 2,490 | 1,580 | 3,924 | 3,931 | 0,000 |
| Connor 2016 | Breast | os | 1,540 | 1,070 | 2,216 | 2,324 | 0,020 |
| Conroy 2011 | Breast | os | 1,540 | 1,230 | 1,928 | 3,765 | 0,000 |
| Copson 2014 | Breast | os | 1,350 | 1,080 | 1,688 | 2,636 | 0,008 |
| Dal Maso 2008 | Breast | os | 1,290 | 0,990 | 1,681 | 1,886 | 0,059 |
| Dawood 2012 | Breast | os | 0,970 | 0,810 | 1,162 | -0,331 | 0,741 |
| Dignam 2003 | Breast | os | 1,310 | 1,120 | 1,532 | 3,377 | 0,001 |
| Dignam 2006 | Breast | os | 1,170 | 0,940 | 1,456 | 1,406 | 0,160 |
| Elwood 2018 | Breast | os | 0,940 | 0,690 | 1,281 | -0,392 | 0,695 |
| Emaus 2009 | Breast | os | 1,470 | 1,080 | 2,001 | 2,449 | 0,014 |
| Goodwin 2012 | Breast | os | 1,190 | 0,890 | 1,591 | 1,174 | 0,241 |
| He 2012 | Breast | os | 0,735 | 0,560 | 0,965 | -2,219 | 0,026 |
| Hellmann 2010 | Breast | os | 1,610 | 1,120 | 2,314 | 2,572 | 0,010 |
| His 2016 | Breast | os | 1,050 | 0,550 | 2,005 | 0,148 | 0,882 |
| Jeon 2015 | Breast | os | 1,290 | 1,130 | 1,473 | 3,769 | 0,000 |
| Jiralerspong 2013 | Breast | os | 1,240 | 1,040 | 1,478 | 2,397 | 0,017 |
| Kawai 2016 | Breast | os | 1,460 | 1,160 | 1,838 | 3,225 | 0,001 |
| Keegan 2010 | Breast | os | 1,210 | 1,000 | 1,464 | 1,960 | 0,050 |
| Kwan 2012 | Breast | os | 1,170 | 1,040 | 1,316 | 2,613 | 0,009 |
| Kwan 2013 | Breast | os | 1,140 | 1,004 | 1,294 | 2,022 | 0,043 |
| Ladoire 2014 | Breast | os | 1,270 | 0,980 | 1,646 | 1,807 | 0,071 |
| Larsen 2015 | Breast | os | 1,430 | 1,060 | 1,929 | 2,341 | 0,019 |
| Loi 2005 | Breast | os | 1,480 | 0,990 | 2,213 | 1,911 | 0,056 |
| Maskarinec 2011 | Breast | os | 2,060 | 1,230 | 3,450 | 2,747 | 0,006 |
| Mazzarella 2013 | Breast | os | 1,450 | 0,940 | 2,237 | 1,680 | 0,093 |
| McCullough 2016 | Breast | os | 1,360 | 1,080 | 1,713 | 2,614 | 0,009 |
| McTiernan 2018 | Breast | os | 1,410 | 1,290 | 1,541 | 7,571 | 0,000 |
| Melhem-Bertrandt 2011 | Breast | os | 1,250 | 0,950 | 1,645 | 1,594 | 0,111 |
| Meyer 2015 | Breast | os | 1,070 | 0,640 | 1,789 | 0,258 | 0,796 |
| Mutschler 2018 | Breast | os | 1,460 | 1,020 | 2,090 | 2,068 | 0,039 |
| Nichols 2009 | Breast | os | 1,520 | 1,170 | 1,975 | 3,136 | 0,002 |
| Nur 2019 | Breast | os | 1,630 | 1,120 | 2,372 | 2,552 | 0,011 |
| Oudanonh 2019 | Breast | os | 1,480 | 1,130 | 1,938 | 2,848 | 0,004 |
| Payares 2013 | Breast | os | 1,140 | 0,730 | 1,780 | 0,576 | 0,565 |
| Pfeiler 2013 | Breast | os | 1,810 | 1,120 | 2,925 | 2,423 | 0,015 |
| Pierce 2007 | Breast | os | 1,420 | 0,870 | 2,318 | 1,403 | 0,161 |
| Probst-Hensch 2010 | Breast | os | 1,040 | 0,470 | 2,301 | 0,097 | 0,923 |
| Sparano 2012 | Breast | os | 1,230 | 1,080 | 1,401 | 3,120 | 0,002 |
| Sparano 2012 (2) | Breast | os | 1,400 | 1,150 | 1,704 | 3,353 | 0,001 |
| Sun 2015 | Breast | os | 1,110 | 0,840 | 1,467 | 0,734 | 0,463 |
| Sun 2018 | Breast | os | 2,050 | 1,260 | 3,335 | 2,891 | 0,004 |
| Taghizadeh 2015 | Breast | os | 1,520 | 0,880 | 2,625 | 1,502 | 0,133 |
| Tait 2014 | Breast | os | 0,865 | 0,550 | 1,360 | -0,628 | 0,530 |
| Widschwendter 2015 | Breast | os | 1,440 | 0,790 | 2,625 | 1,190 | 0,234 |
| Xiao 2014 | Breast | os | 1,006 | 0,780 | 1,297 | 0,046 | 0,963 |
| Yoon 2011 | Breast | os | 1,110 | 0,870 | 1,416 | 0,840 | 0,401 |
| Boyle 2013 | CRC | os | 1,260 | 0,880 | 1,804 | 1,262 | 0,207 |
| Campbell 2015 | CRC | os | 1,300 | 1,180 | 1,432 | 5,310 | 0,000 |
| Cespedes Feliciano 2017 | CRC | os | 2,540 | 1,710 | 3,773 | 4,617 | 0,000 |
| Clark 2013 | CRC | os | 1,770 | 0,590 | 5,310 | 1,019 | 0,308 |
| Dahdaleh 2018 | CRC | os | 1,750 | 1,220 | 2,510 | 3,040 | 0,002 |
| Dignam 2006 | CRC | os | 1,190 | 1,000 | 1,416 | 1,960 | 0,050 |
| Doria-Rose 2006 | CRC | os | 1,500 | 1,000 | 2,250 | 1,960 | 0,050 |
| Fedirko 2014 | CRC | os | 1,320 | 1,120 | 1,556 | 3,312 | 0,001 |
| Jayasekara 2018 | CRC | os | 1,140 | 0,820 | 1,585 | 0,779 | 0,436 |
| Kaidar-Person 2015 | CRC | os | 0,620 | 0,330 | 1,165 | -1,486 | 0,137 |
| Kalb 2019 | CRC | os | 0,930 | 0,460 | 1,880 | -0,202 | 0,840 |
| Kocarnik 2017 | CRC | os | 1,690 | 1,000 | 2,856 | 1,960 | 0,050 |
| Mayerhardt 2008 | CRC | os | 0,885 | 0,575 | 1,362 | -0,555 | 0,579 |
| Meyer 2015 | CRC | os | 1,300 | 0,870 | 1,943 | 1,280 | 0,200 |
| Meyerhardt 2003 | CRC | os | 1,340 | 1,070 | 1,678 | 2,549 | 0,011 |
| Meyerhardt 2004 | CRC | os | 1,090 | 0,900 | 1,320 | 0,882 | 0,378 |
| Meyerhardt/2008 | CRC | os | 0,945 | 0,575 | 1,553 | -0,223 | 0,823 |
| Morikawa 2012 | CRC | os | 1,170 | 0,930 | 1,472 | 1,340 | 0,180 |
| Ogino 2009 | CRC | os | 1,170 | 0,440 | 3,111 | 0,315 | 0,753 |
| Patel 2015 | CRC | os | 1,070 | 0,880 | 1,301 | 0,678 | 0,498 |
| Pelser 2014 | CRC | os | 1,190 | 1,020 | 1,388 | 2,212 | 0,027 |
| Prizment 2010 | CRC | os | 1,460 | 1,150 | 1,854 | 3,108 | 0,002 |
| Schlesinger 2014 | CRC | os | 0,840 | 0,620 | 1,138 | -1,125 | 0,260 |
| Shah 2015 | CRC | os | 1,090 | 0,710 | 1,673 | 0,394 | 0,694 |
| Sinicrope 2012 | CRC | os | 1,340 | 1,120 | 1,603 | 3,198 | 0,001 |
| Sinicrope 2013 | CRC | os | 1,100 | 1,040 | 1,163 | 3,330 | 0,001 |
| Taghizadeh 2015 | CRC | os | 1,280 | 0,730 | 2,244 | 0,862 | 0,389 |
| Wang 2017 | CRC | os | 1,540 | 1,120 | 2,118 | 2,657 | 0,008 |
| Wang 2019 | CRC | os | 0,590 | 0,350 | 0,995 | -1,980 | 0,048 |
| You 2015 | CRC | os | 1,470 | 0,080 | 27,011 | 0,259 | 0,795 |
| Zheng 2015 | CRC | os | 1,440 | 0,880 | 2,356 | 1,451 | 0,147 |
| Hynes 2017 | Gastroesophageal | os | 0,870 | 0,580 | 1,305 | -0,673 | 0,501 |
| Lee 2018 | Gastroesophageal | os | 0,770 | 0,510 | 1,163 | -1,243 | 0,214 |
| Meyer 2015 | Gastroesophageal | os | 1,450 | 0,710 | 2,961 | 1,020 | 0,308 |
| Spreafico 2017 | Gastroesophageal | os | 2,630 | 1,810 | 3,821 | 5,072 | 0,000 |
| Sudelof 2008 | Gastroesophageal | os | 0,970 | 0,660 | 1,426 | -0,155 | 0,877 |
| Thrift 2012 | Gastroesophageal | os | 0,970 | 0,640 | 1,470 | -0,144 | 0,886 |
| Trivers 2005 | Gastroesophageal | os | 0,830 | 0,640 | 1,076 | -1,405 | 0,160 |
| Gama 2017 | Head & Neck | os | 0,790 | 0,500 | 1,248 | -1,010 | 0,312 |
| Grossberg 2016 | Head & Neck | os | 0,300 | 0,150 | 0,600 | -3,404 | 0,001 |
| Meyer 2015 | Head & Neck | os | 0,780 | 0,420 | 1,449 | -0,787 | 0,431 |
| Ata 2018 | Hepatobiliary | os | 0,930 | 0,820 | 1,055 | -1,130 | 0,259 |
| Carr 2018 | Hepatobiliary | os | 0,840 | 0,520 | 1,357 | -0,713 | 0,476 |
| Mathur 2010 | Hepatobiliary | os | 0,960 | 0,920 | 1,002 | -1,880 | 0,060 |
| Meyer 2015 | Hepatobiliary | os | 2,210 | 1,180 | 4,139 | 2,477 | 0,013 |
| Yang 2019 | Hepatobiliary | os | 1,410 | 1,080 | 1,841 | 2,526 | 0,012 |
| Abdel-Rahman 2019 | Lung | os | 0,780 | 0,690 | 0,882 | -3,972 | 0,000 |
| Aldrich 2013 | Lung | os | 0,920 | 0,690 | 1,227 | -0,568 | 0,570 |
| Kichenadasse 2019 | Lung | os | 0,780 | 0,600 | 1,014 | -1,856 | 0,063 |
| Meyer 2015 | Lung | os | 0,800 | 0,570 | 1,123 | -1,290 | 0,197 |
| Nagakawa 2016 | Lung | os | 0,480 | 0,170 | 1,355 | -1,386 | 0,166 |
| Ozyurek 2018 | Lung | os | 1,280 | 0,620 | 2,643 | 0,667 | 0,504 |
| Qi 2009 | Lung | os | 0,780 | 0,580 | 1,049 | -1,644 | 0,100 |
| Sepesi 2017 | Lung | os | 0,800 | 0,670 | 0,955 | -2,466 | 0,014 |
| Shepshelovich 2019 | Lung | os | 0,960 | 0,880 | 1,047 | -0,920 | 0,358 |
| Taghizadeh 2015 | Lung | os | 0,780 | 0,470 | 1,294 | -0,961 | 0,336 |
| Xie 2017 | Lung | os | 2,870 | 1,480 | 5,565 | 3,120 | 0,002 |
| McQuade 2018 | Melanoma | os | 0,740 | 0,630 | 0,869 | -3,667 | 0,000 |
| Bandera 2015 | Ovarian | os | 0,680 | 0,430 | 1,075 | -1,649 | 0,099 |
| Minlikeeva 2019 | Ovarian | os | 1,160 | 1,070 | 1,258 | 3,602 | 0,000 |
| Previs 2014 | Ovarian | os | 2,080 | 0,930 | 4,652 | 1,783 | 0,075 |
| Tyler 2012 | Ovarian | os | 0,900 | 0,600 | 1,350 | -0,509 | 0,611 |
| Dalal 2012 | Pancreas | os | 2,680 | 1,200 | 5,985 | 2,405 | 0,016 |
| Gong 2012 | Pancreas | os | 1,280 | 0,910 | 1,800 | 1,418 | 0,156 |
| Li 2009 | Pancreas | os | 1,860 | 1,350 | 2,563 | 3,795 | 0,000 |
| Meyer 2015 | Pancreas | os | 1,600 | 0,930 | 2,753 | 1,698 | 0,090 |
| Olson 2010 | Pancreas | os | 1,230 | 0,890 | 1,700 | 1,254 | 0,210 |
| Tsai 2010 | Pancreas | os | 0,730 | 0,560 | 0,952 | -2,327 | 0,020 |
| Bonn 2014 | Prostate | os | 1,470 | 1,030 | 2,098 | 2,123 | 0,034 |
| Efstathiou 2008 | Prostate | os | 1,000 | 0,750 | 1,333 | 0,000 | 1,000 |
| Farris 2018 | Prostate | os | 1,090 | 0,880 | 1,350 | 0,789 | 0,430 |
| Froehner 2014 | Prostate | os | 1,470 | 1,100 | 1,964 | 2,604 | 0,009 |
| Halabi 2007 | Prostate | os | 0,800 | 0,680 | 0,941 | -2,691 | 0,007 |
| Han 2010 | Prostate | os | 1,160 | 0,820 | 1,641 | 0,839 | 0,402 |
| Meyer 2015 | Prostate | os | 1,540 | 0,930 | 2,550 | 1,678 | 0,093 |
| Montgomery 2007 | Prostate | os | 0,900 | 0,720 | 1,125 | -0,925 | 0,355 |
| Rudman 2016 | Prostate | os | 1,060 | 0,700 | 1,605 | 0,275 | 0,783 |
| Taghizadeh 2015 | Prostate | os | 3,330 | 1,310 | 8,465 | 2,527 | 0,011 |
| Vidal 2018 | Prostate | os | 0,790 | 0,650 | 0,960 | -2,369 | 0,018 |
| Wu 2015 | Prostate | os | 0,670 | 0,304 | 1,474 | -0,995 | 0,320 |
| Keizman 2014 | RCC | os | 0,890 | 0,540 | 1,467 | -0,457 | 0,648 |
| Parker 2006 | RCC | os | 0,720 | 0,570 | 0,909 | -2,756 | 0,006 |
| Psutka 2015 | RCC | os | 1,250 | 0,870 | 1,796 | 1,207 | 0,228 |
| Spiess 2012 | RCC | os | 0,420 | 0,220 | 0,802 | -2,629 | 0,009 |
| Yu 1991 | RCC | os | 0,680 | 0,380 | 1,217 | -1,299 | 0,194 |
| Sanchez 2019 | RCC | os | 0,602 | 0,461 | 0,786 | -3,727 | 0,000 |
| Arem 2013 | Utherine | os | 1,950 | 1,500 | 2,535 | 4,989 | 0,000 |
| Frumovitz 2014 | Utherine | os | 1,260 | 1,100 | 1,443 | 3,336 | 0,001 |
| Kristensen 2017 | Utherine | os | 1,220 | 1,020 | 1,459 | 2,177 | 0,030 |
| Matsuo 2016 | Utherine | os | 1,190 | 0,620 | 2,284 | 0,523 | 0,601 |
| Meyer 2015 | Utherine | os | 1,880 | 1,100 | 3,213 | 2,309 | 0,021 |
| Nagle 2018 | Utherine | os | 1,210 | 0,880 | 1,664 | 1,173 | 0,241 |
| Nicholas 2014 | Utherine | os | 1,400 | 0,860 | 2,279 | 1,353 | 0,176 |
| Roque 2016 | Utherine | os | 0,580 | 0,340 | 0,989 | -1,999 | 0,046 |
| Ruterbusch 2014 | Utherine | os | 1,100 | 0,830 | 1,458 | 0,663 | 0,507 |
| Seidelin 2016 | Utherine | os | 1,120 | 0,940 | 1,334 | 1,268 | 0,205 |
| Todo 2014 | Utherine | os | 0,880 | 0,510 | 1,518 | -0,459 | 0,646 |
| Yoon 2015 | Utherine | os | 0,780 | 0,450 | 1,352 | -0,885 | 0,376 |
| Cortellini 2019 | Various | os | 0,500 | 0,320 | 0,781 | -3,044 | 0,002 |
| Hung 2018 | Various | os | 0,480 | 0,390 | 0,591 | -6,928 | 0,000 |
| Janssen 2015 | Various | os | 1,046 | 0,540 | 2,026 | 0,133 | 0,894 |
| Jenkins 2018 | Various | os | 1,460 | 1,330 | 1,603 | 7,953 | 0,000 |
| Kitahara 2014 | Various | os | 2,570 | 2,140 | 3,086 | 10,104 | 0,000 |
| Martini 2020 | Various | os | 0,540 | 0,250 | 1,166 | -1,568 | 0,117 |
| Meyer 2015 | Various | os | 1,080 | 0,800 | 1,458 | 0,503 | 0,615 |
| Parr 2010 | Various | os | 0,970 | 0,910 | 1,034 | -0,935 | 0,350 |
| Yano 2013 | Various | os | 1,180 | 0,730 | 1,907 | 0,676 | 0,499 |
| | | | 1,144 | 1,097 | 1,193 | 6,335 | 0,000 |

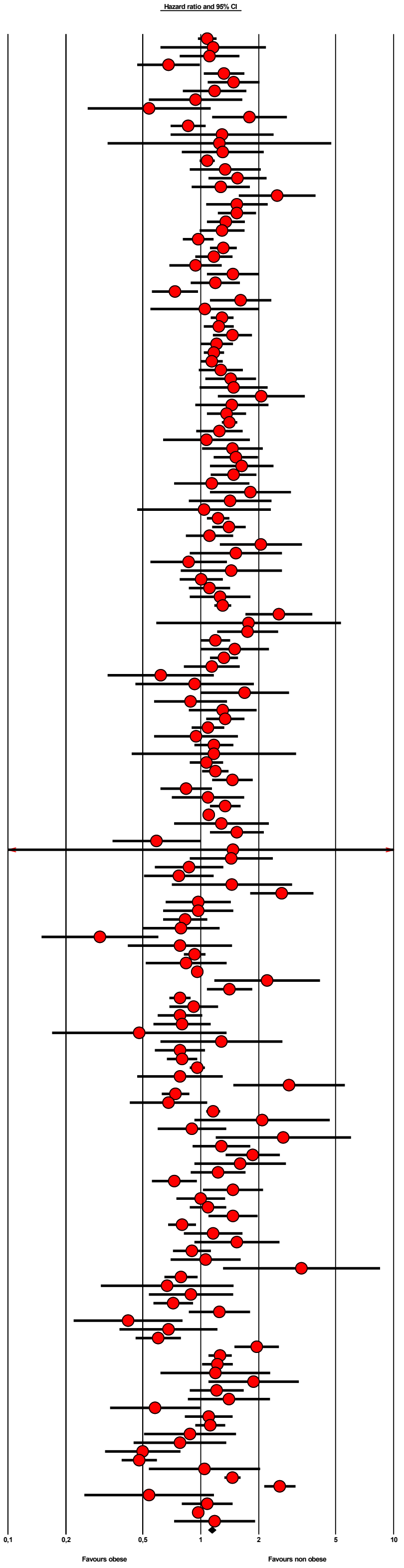


© 2021 Petrelli F et al. *JAMA Network Open.*

**eFigure 2.** Forest Plot for Cancer-Specific Survival

| Study name | Subgroup within study | Outcome | Statistics for each study | | | | |
|---|---|---|---|---|---|---|---|
| | | | Hazard ratio | Lower limit | Upper limit | Z-Value | p-Value |
| Chromecki 2012 | Bladder/UTUC | css | 1,260 | 1,100 | 1,443 | 3,336 | 0,001 |
| Murakami 2018 | Bladder/UTUC | css | 3,460 | 1,380 | 8,675 | 2,647 | 0,008 |
| Xu 2018 | Bladder/UTUC | css | 1,130 | 0,770 | 1,658 | 0,624 | 0,532 |
| Alsaker 2011 | Breast | css | 1,520 | 1,250 | 1,848 | 4,196 | 0,000 |
| Beasley 2012 | Breast | css | 0,720 | 0,610 | 0,850 | -3,884 | 0,000 |
| Blair 2019 | Breast | css | 1,330 | 0,950 | 1,862 | 1,661 | 0,097 |
| Braithwaite 2010 | Breast | css | 0,720 | 0,340 | 1,525 | -0,858 | 0,391 |
| Caan 2008 | Breast | css | 1,200 | 0,700 | 2,057 | 0,663 | 0,507 |
| Cespedes-Feliciano 2017 | Breast | css | 1,010 | 0,790 | 1,291 | 0,079 | 0,937 |
| Chen 2010 | Breast | css | 1,440 | 1,020 | 2,033 | 2,073 | 0,038 |
| Chung 2017 | Breast | css | 1,200 | 0,750 | 1,920 | 0,760 | 0,447 |
| Cleveland 2011 | Breast | css | 1,220 | 0,510 | 2,918 | 0,447 | 0,655 |
| Connor 2016 | Breast | css | 0,970 | 0,570 | 1,651 | -0,112 | 0,911 |
| Conroy 2011 | Breast | css | 1,450 | 1,050 | 2,002 | 2,256 | 0,024 |
| Dal Maso 2008 | Breast | css | 1,380 | 1,020 | 1,867 | 2,088 | 0,037 |
| Dignam 2003 | Breast | css | 1,200 | 0,970 | 1,485 | 1,679 | 0,093 |
| Dignam 2006 | Breast | css | 1,070 | 0,820 | 1,396 | 0,498 | 0,618 |
| Elwood 2018 | Breast | css | 0,980 | 0,690 | 1,392 | -0,113 | 0,910 |
| Emaus 2009 | Breast | css | 1,430 | 1,010 | 2,025 | 2,016 | 0,044 |
| Han 2014 | Breast | css | 1,240 | 0,940 | 1,636 | 1,522 | 0,128 |
| Hellmann 2010 | Breast | css | 1,820 | 1,110 | 2,984 | 2,374 | 0,018 |
| His 2016 | Breast | css | 1,720 | 0,790 | 3,745 | 1,366 | 0,172 |
| Jeon 2015 | Breast | css | 1,210 | 1,020 | 1,435 | 2,187 | 0,029 |
| Jiralerspong 2013 | Breast | css | 1,230 | 1,000 | 1,513 | 1,960 | 0,050 |
| Kawai 2016 | Breast | css | 1,470 | 1,110 | 1,947 | 2,688 | 0,007 |
| Kwan 2012 | Breast | css | 1,100 | 0,950 | 1,274 | 1,274 | 0,203 |
| Kwan 2013 | Breast | css | 1,100 | 0,970 | 1,247 | 1,485 | 0,137 |
| Maskarinec 2011 | Breast | css | 2,990 | 1,220 | 7,328 | 2,395 | 0,017 |
| McCullough 2005 | Breast | css | 1,470 | 1,130 | 1,912 | 2,871 | 0,004 |
| McCullough 2016 | Breast | css | 1,630 | 1,150 | 2,310 | 2,745 | 0,006 |
| McTiernan 2018 | Breast | css | 1,350 | 1,240 | 1,470 | 6,920 | 0,000 |
| Nichols 2009 | Breast | css | 1,420 | 0,860 | 2,345 | 1,370 | 0,171 |
| Nur 2019 | Breast | css | 1,450 | 0,810 | 2,596 | 1,251 | 0,211 |
| Oudanonh 2019 | Breast | css | 1,330 | 0,910 | 1,944 | 1,473 | 0,141 |
| Pajares 2013 | Breast | css | 1,130 | 0,940 | 1,358 | 1,301 | 0,193 |
| Rosenberg 2009 | Breast | css | 1,400 | 1,100 | 1,782 | 2,735 | 0,006 |
| Sparano 2012 (2) | Breast | css | 1,450 | 1,160 | 1,813 | 3,264 | 0,001 |
| Sun 2015 | Breast | css | 0,970 | 0,680 | 1,384 | -0,168 | 0,867 |
| Yoon 2011 | Breast | css | 1,140 | 0,880 | 1,477 | 0,992 | 0,321 |
| Boyle 2013 | CRC | css | 1,330 | 0,890 | 1,988 | 1,391 | 0,164 |
| Campbell 2015 | CRC | css | 1,200 | 1,060 | 1,358 | 2,881 | 0,004 |
| Cespedes Feliciano 2017 | CRC | css | 2,290 | 1,350 | 3,885 | 3,073 | 0,002 |
| Dignam 2006 | CRC | css | 1,220 | 0,980 | 1,519 | 1,779 | 0,075 |
| Doria-Rose 2006 | CRC | css | 1,500 | 0,900 | 2,500 | 1,556 | 0,120 |
| Fedirko 2014 | CRC | css | 1,260 | 1,040 | 1,527 | 2,361 | 0,018 |
| Jayasekara 2018 | CRC | css | 1,410 | 0,900 | 2,209 | 1,500 | 0,134 |
| Kocarnik 2017 | CRC | css | 1,860 | 0,670 | 5,164 | 1,191 | 0,234 |
| Morikawa 2012 | CRC | css | 1,100 | 0,810 | 1,494 | 0,610 | 0,542 |
| Ogino 2009 | CRC | css | 1,100 | 0,240 | 5,042 | 0,123 | 0,902 |
| Pelser 2014 | CRC | css | 1,150 | 0,960 | 1,378 | 1,517 | 0,129 |
| Prizment 2010 | CRC | css | 1,300 | 0,950 | 1,779 | 1,639 | 0,101 |
| Wang 2017 | CRC | css | 1,510 | 1,080 | 2,111 | 2,410 | 0,016 |
| Hynes 2017 | Gastroesophageal | css | 0,890 | 0,570 | 1,390 | -0,513 | 0,608 |
| Lee 2018 | Gastroesophageal | css | 0,750 | 0,440 | 1,278 | -1,057 | 0,290 |
| Grossberg 2016 | Head & Neck | css | 0,260 | 0,110 | 0,615 | -3,069 | 0,002 |
| Hu 2019 | Head & Neck | css | 2,022 | 1,040 | 3,931 | 2,076 | 0,038 |
| Iyengar 2014 | Head & Neck | css | 5,010 | 1,690 | 14,852 | 2,906 | 0,004 |
| McMahon 2017 | Hepatobiliary | css | 0,790 | 0,500 | 1,248 | -1,010 | 0,312 |
| Leung 2010 | Lung | css | 0,550 | 0,100 | 3,025 | -0,687 | 0,492 |
| Nonemaker 2009 | Lung | css | 0,500 | 0,160 | 1,563 | -1,192 | 0,233 |
| Turner 2011 | Lung | css | 0,540 | 0,270 | 1,080 | -1,742 | 0,081 |
| Bandera 2015 | Ovarian | css | 0,670 | 0,400 | 1,122 | -1,522 | 0,128 |
| Kotsopoulos 2012 | Ovarian | css | 1,110 | 0,870 | 1,416 | 0,840 | 0,401 |
| Previs 2014 | Ovarian | css | 1,660 | 0,570 | 4,834 | 0,929 | 0,353 |
| Yang 2008 | Ovarian | css | 1,230 | 0,870 | 1,739 | 1,172 | 0,241 |
| Genkinger 2015 | Pancreas | css | 1,480 | 1,270 | 1,725 | 5,021 | 0,000 |
| Lin 2013 | Pancreas | css | 0,960 | 0,670 | 1,376 | -0,222 | 0,824 |
| Yuan 2013 | Pancreas | css | 1,280 | 1,060 | 1,546 | 2,566 | 0,010 |
| Bassett 2011 | Prostate | css | 1,520 | 0,890 | 2,596 | 1,533 | 0,125 |
| Bonn 2014 | Prostate | css | 1,290 | 0,670 | 2,484 | 0,762 | 0,446 |
| Dickerman 2017 | Prostate | css | 1,090 | 0,760 | 1,563 | 0,468 | 0,640 |
| Efstathiou 2008 | Prostate | css | 1,640 | 1,010 | 2,663 | 2,000 | 0,045 |
| Farris 2018 | Prostate | css | 1,130 | 0,800 | 1,596 | 0,694 | 0,488 |
| Gong 2007 | Prostate | css | 2,640 | 1,180 | 5,906 | 2,363 | 0,018 |
| Halabi 2007 | Prostate | css | 0,810 | 0,680 | 0,965 | -2,361 | 0,018 |
| Kelly 2017 | Prostate | css | 1,460 | 1,020 | 2,090 | 2,068 | 0,039 |
| Kenfield 2016 | Prostate | css | 1,070 | 0,818 | 1,400 | 0,493 | 0,622 |
| Khan 2017 | Prostate | css | 1,400 | 0,990 | 1,980 | 1,903 | 0,057 |
| Ma 2008 | Prostate | css | 1,950 | 1,170 | 3,250 | 2,562 | 0,010 |
| Moller 2014 | Prostate | css | 1,270 | 0,900 | 1,792 | 1,360 | 0,174 |
| Rudman 2016 | Prostate | css | 1,020 | 0,550 | 1,892 | 0,063 | 0,950 |
| Vidal 2017 | Prostate | css | 2,050 | 1,040 | 4,041 | 2,073 | 0,038 |
| Vidal 2018 | Prostate | css | 1,110 | 0,860 | 1,433 | 0,802 | 0,423 |
| Eskelinen 2017 | RCC | css | 1,020 | 0,770 | 1,351 | 0,138 | 0,890 |
| Liss 2016 | RCC | css | 2,840 | 1,290 | 6,252 | 2,592 | 0,010 |
| Parker 2006 | RCC | css | 0,480 | 0,360 | 0,640 | -5,000 | 0,000 |
| Psutka 2015 | RCC | css | 1,310 | 0,800 | 2,145 | 1,073 | 0,283 |
| Arem 2013 | Utherine | css | 1,790 | 1,200 | 2,670 | 2,854 | 0,004 |
| Frumovitz 2014 | Utherine | css | 1,240 | 1,060 | 1,451 | 2,688 | 0,007 |
| Matsuo 2018 | Utherine | css | 0,220 | 0,060 | 0,807 | -2,284 | 0,022 |
| Nagle 2018 | Utherine | css | 0,840 | 0,560 | 1,260 | -0,843 | 0,399 |
| Ruterbusch 2014 | Utherine | css | 0,900 | 0,580 | 1,397 | -0,470 | 0,638 |
| Todo 2014 | Utherine | css | 0,880 | 0,510 | 1,518 | -0,459 | 0,646 |
| Abdullah 2011 | Various | css | 1,030 | 1,010 | 1,050 | 2,955 | 0,003 |
| Akinyemiju 2018 | Various | css | 0,760 | 0,610 | 0,947 | -2,447 | 0,014 |
| Barroso 2017 | Various | css | 3,550 | 0,530 | 23,778 | 1,306 | 0,192 |
| Boggs 2011 | Various | css | 1,120 | 0,860 | 1,459 | 0,841 | 0,400 |
| Drake 2017 | Various | css | 1,450 | 1,170 | 1,797 | 3,394 | 0,001 |
| Han 2014 | Various | css | 1,290 | 0,980 | 1,698 | 1,816 | 0,069 |
| Izumida 2019 | Various | css | 2,720 | 1,340 | 5,521 | 2,770 | 0,006 |
| Katzmarzyk 2012 | Various | css | 1,490 | 1,190 | 1,866 | 3,476 | 0,001 |
| Landman 2008 | Various | css | 0,570 | 0,320 | 1,015 | -1,908 | 0,056 |
| Nechuta 2010 | Various | css | 0,820 | 0,620 | 1,085 | -1,391 | 0,164 |
| Sasazuki 2011 | Various | css | 1,250 | 1,070 | 1,460 | 2,813 | 0,005 |
| Silventoinen 2014 | Various | css | 1,540 | 1,270 | 1,867 | 4,390 | 0,000 |
| Tseng 2013 | Various | css | 0,830 | 0,730 | 0,944 | -2,845 | 0,004 |
| Tseng 2016 | Various | css | 1,012 | 0,918 | 1,116 | 0,240 | 0,810 |
| Valentijn 2013 | Various | css | 0,630 | 0,460 | 0,863 | -2,879 | 0,004 |
| Xu 2018 | Various | css | 1,150 | 0,970 | 1,363 | 1,609 | 0,108 |
| | | | 1,179 | 1,125 | 1,236 | 6,856 | 0,000 |

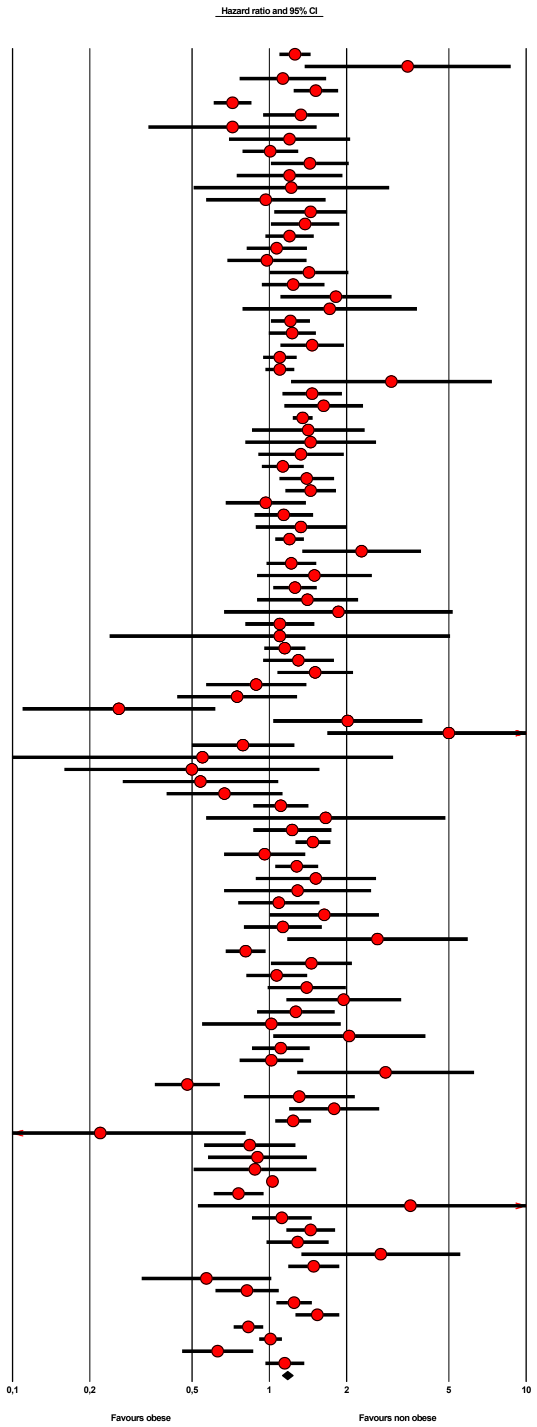


© 2021 Petrelli F et al. *JAMA Network Open.*

# **eFigure 3.** Forest Plot for Disease-Free Survival

| Study name | Subgroup within study | Outcome | Statistics for each study | | | | |
|---|---|---|---|---|---|---|---|
| | | | Hazard ratio | Lower limit | Upper limit | Z-Value | p-Value |
| Chromecki 2012 | Bladder/UTUC | pfs | 1,160 | 1,020 | 1,319 | 2,262 | 0,024 |
| Ferro 2018 | Bladder/UTUC | pfs | 2,510 | 1,760 | 3,580 | 5,081 | 0,000 |
| Xu 2018 | Bladder/UTUC | pfs | 1,060 | 0,780 | 1,441 | 0,372 | 0,710 |
| Abubakar 2018 | Breast | pfs | 1,180 | 0,690 | 2,018 | 0,605 | 0,545 |
| Ademuyiwa 2011 | Breast | pfs | 0,810 | 0,490 | 1,339 | -0,822 | 0,411 |
| Beasley 2012 | Breast | pfs | 0,960 | 0,770 | 1,197 | -0,363 | 0,717 |
| Buono 2017 | Breast | pfs | 1,510 | 0,570 | 4,000 | 0,829 | 0,407 |
| Caan 2008 | Breast | pfs | 1,000 | 0,700 | 1,429 | 0,000 | 1,000 |
| Cespedes-Feliciano 2017 | Breast | pfs | 0,990 | 0,750 | 1,307 | -0,071 | 0,943 |
| Copson 2014 | Breast | pfs | 1,210 | 1,000 | 1,464 | 1,960 | 0,050 |
| Crozier 2013 | Breast | pfs | 1,280 | 1,050 | 1,560 | 2,443 | 0,015 |
| Dawood 2012 | Breast | pfs | 1,020 | 0,860 | 1,210 | 0,227 | 0,820 |
| Dignam 2003 | Breast | pfs | 0,980 | 0,800 | 1,201 | -0,195 | 0,845 |
| Dignam 2006 | Breast | pfs | 1,170 | 0,980 | 1,397 | 1,737 | 0,082 |
| Elwood 2018 | Breast | pfs | 1,300 | 0,810 | 2,086 | 1,087 | 0,277 |
| Goodwin 2012 | Breast | pfs | 1,150 | 0,850 | 1,556 | 0,906 | 0,365 |
| His 2016 | Breast | pfs | 0,920 | 0,570 | 1,485 | -0,341 | 0,733 |
| Jiralerspong 2013 | Breast | pfs | 1,130 | 0,980 | 1,303 | 1,682 | 0,093 |
| Kawai 2016 | Breast | pfs | 1,150 | 0,950 | 1,392 | 1,434 | 0,152 |
| Kwan 2012 | Breast | pfs | 1,060 | 0,940 | 1,195 | 0,951 | 0,342 |
| Ladoire 2014 | Breast | pfs | 1,130 | 0,930 | 1,373 | 1,230 | 0,219 |
| Loi 2005 | Breast | pfs | 1,500 | 1,070 | 2,103 | 2,353 | 0,019 |
| Mazzarella 2013 | Breast | pfs | 1,050 | 0,730 | 1,510 | 0,263 | 0,792 |
| Melhem-Bertrandt 2011 | Breast | pfs | 1,160 | 0,900 | 1,495 | 1,146 | 0,252 |
| Mutschler 2018 | Breast | pfs | 1,380 | 1,030 | 1,849 | 2,158 | 0,031 |
| Oh 2011 | Breast | pfs | 1,020 | 0,505 | 2,060 | 0,055 | 0,956 |
| Pajares 2013 | Breast | pfs | 1,130 | 0,980 | 1,303 | 1,682 | 0,093 |
| Pfeiler 2013 | Breast | pfs | 1,450 | 1,030 | 2,041 | 2,129 | 0,033 |
| Senie 1992 | Breast | pfs | 1,290 | 1,000 | 1,664 | 1,960 | 0,050 |
| Sparano 2012 | Breast | pfs | 1,170 | 1,040 | 1,316 | 2,613 | 0,009 |
| Sparano 2012 (2) | Breast | pfs | 1,240 | 1,080 | 1,424 | 3,052 | 0,002 |
| Su 2013 | Breast | pfs | 1,000 | 0,740 | 1,351 | 0,000 | 1,000 |
| Sun 2018 | Breast | pfs | 1,880 | 1,280 | 2,761 | 3,219 | 0,001 |
| Tait 2014 | Breast | pfs | 0,945 | 0,605 | 1,476 | -0,249 | 0,804 |
| Warren 2016 | Breast | pfs | 1,440 | 0,970 | 2,138 | 1,809 | 0,070 |
| Widschwendter 2015 | Breast | pfs | 1,380 | 0,760 | 2,506 | 1,058 | 0,290 |
| Yoon 2011 | Breast | pfs | 1,210 | 0,960 | 1,525 | 1,614 | 0,106 |
| Clark 2013 | CRC | pfs | 1,520 | 0,630 | 3,667 | 0,932 | 0,351 |
| Dahdaleh 2018 | CRC | pfs | 1,670 | 1,290 | 2,162 | 3,893 | 0,000 |
| Kaidar-Person 2015 | CRC | pfs | 0,940 | 0,680 | 1,299 | -0,375 | 0,708 |
| Mayerhardt 2008 | CRC | pfs | 1,120 | 0,760 | 1,651 | 0,573 | 0,567 |
| Meyerhardt 2003 | CRC | pfs | 1,240 | 0,980 | 1,569 | 1,792 | 0,073 |
| Meyerhardt 2004 | CRC | pfs | 1,100 | 0,910 | 1,330 | 0,985 | 0,325 |
| Meyerhardt/2008 | CRC | dfs | 1,120 | 0,780 | 1,608 | 0,614 | 0,539 |
| Sinicrope 2012 | CRC | pfs | 1,370 | 1,140 | 1,646 | 3,357 | 0,001 |
| Sinicrope 2013 | CRC | pfs | 1,060 | 1,000 | 1,124 | 1,960 | 0,050 |
| Sorbye 2012 | CRC | pfs | 1,040 | 0,750 | 1,442 | 0,235 | 0,814 |
| Wang 2019 | CRC | pfs | 0,610 | 0,420 | 0,886 | -2,596 | 0,009 |
| You 2015 | CRC | pfs | 1,030 | 0,730 | 1,453 | 0,168 | 0,866 |
| Zheng 2015 | CRC | pfs | 1,590 | 1,140 | 2,218 | 2,732 | 0,006 |
| Spreafico 2017 | Gastroesophageal | pfs | 1,620 | 1,130 | 2,322 | 2,625 | 0,009 |
| Gama 2017 | Head & Neck | pfs | 1,100 | 0,700 | 1,729 | 0,413 | 0,679 |
| Grossberg 2016 | Head & Neck | pfs | 0,390 | 0,150 | 1,014 | -1,931 | 0,053 |
| Hu 2019 | Head & Neck | pfs | 2,016 | 1,100 | 3,695 | 2,268 | 0,023 |
| Mathur 2010 | Hepatobiliary | pfs | 0,900 | 0,860 | 0,942 | -4,542 | 0,000 |
| Yang 2019 | Hepatobiliary | pfs | 1,310 | 1,030 | 1,666 | 2,201 | 0,028 |
| Kichenadasse 2019 | Lung | pfs | 0,860 | 0,730 | 1,013 | -1,804 | 0,071 |
| Nagakawa 2016 | Lung | pfs | 0,280 | 0,100 | 0,784 | -2,423 | 0,015 |
| McQuade 2018 | Melanoma | pfs | 0,790 | 0,690 | 0,904 | -3,414 | 0,001 |
| Minlikeeva 2019 | Ovarian | pfs | 1,040 | 0,920 | 1,176 | 0,627 | 0,531 |
| Previs 2014 | Ovarian | pfs | 1,470 | 0,080 | 27,011 | 0,259 | 0,795 |
| Bonn 2014 | Prostate | pfs | 1,110 | 0,810 | 1,521 | 0,649 | 0,516 |
| Cullen 2017 | Prostate | pfs | 1,390 | 0,970 | 1,992 | 1,794 | 0,073 |
| Gong 2007 | Prostate | pfs | 3,610 | 1,730 | 7,533 | 3,420 | 0,001 |
| Halabi 2007 | Prostate | pfs | 0,890 | 0,760 | 1,042 | -1,446 | 0,148 |
| Ho 2012 | Prostate | pfs | 1,730 | 1,120 | 2,672 | 2,471 | 0,013 |
| Maj-Hes 2017 | Prostate | pfs | 2,150 | 1,710 | 2,703 | 6,552 | 0,000 |
| Montgomery 2007 | Prostate | pfs | 1,000 | 0,800 | 1,250 | 0,000 | 1,000 |
| Schiffmann 2017 | Prostate | pfs | 0,980 | 0,860 | 1,117 | -0,303 | 0,762 |
| Spangler 2007 | Prostate | pfs | 1,790 | 1,290 | 2,484 | 3,484 | 0,000 |
| Vidal 2017 | Prostate | pfs | 1,110 | 0,940 | 1,311 | 1,230 | 0,219 |
| Vidal 2018 | Prostate | pfs | 1,060 | 0,840 | 1,338 | 0,491 | 0,623 |
| Keizman 2014 | RCC | pfs | 0,810 | 0,530 | 1,238 | -0,974 | 0,330 |
| Lee 2010 | RCC | pfs | 0,215 | 0,053 | 0,872 | -2,151 | 0,031 |
| Psutka 2015 | RCC | pfs | 1,090 | 0,740 | 1,606 | 0,436 | 0,663 |
| Yu 1991 | RCC | pfs | 0,430 | 0,190 | 0,973 | -2,025 | 0,043 |
| Houdek 2019 | Sarcoma | pfs | 0,890 | 0,470 | 1,685 | -0,358 | 0,721 |
| Matsuo 2016 | Utherine | pfs | 1,440 | 0,860 | 2,411 | 1,386 | 0,166 |
| Roque 2016 | Utherine | pfs | 0,660 | 0,370 | 1,177 | -1,407 | 0,159 |
| Cortellini 2019 | Various | pfs | 0,720 | 0,490 | 1,058 | -1,673 | 0,094 |
| | | | 1,136 | 1,079 | 1,195 | 4,887 | 0,000 |

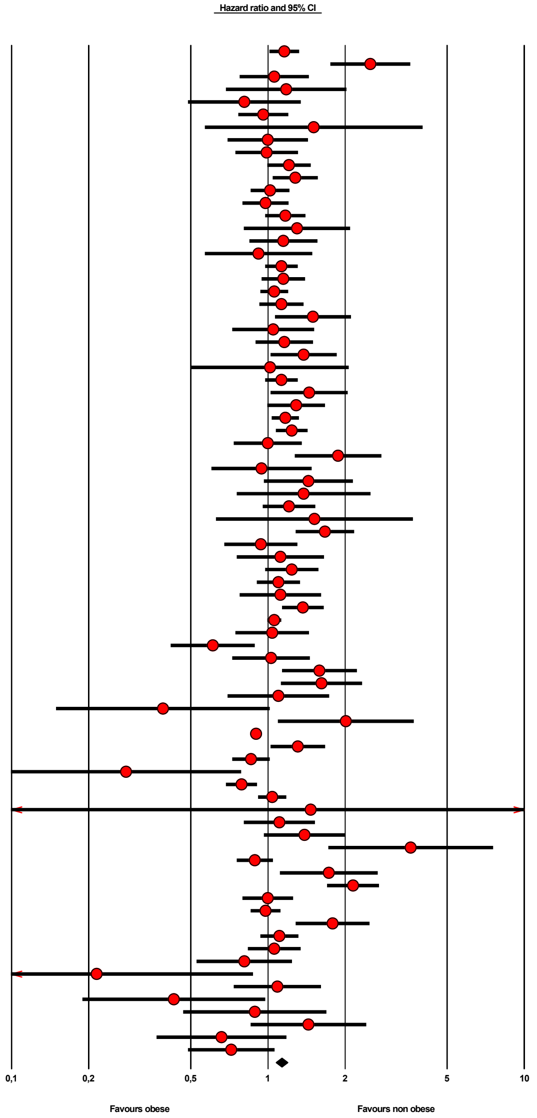


© 2021 Petrelli F et al. *JAMA Network Open.*

**eFigure 4.** Funnel Plot for Overall Survival Analysis

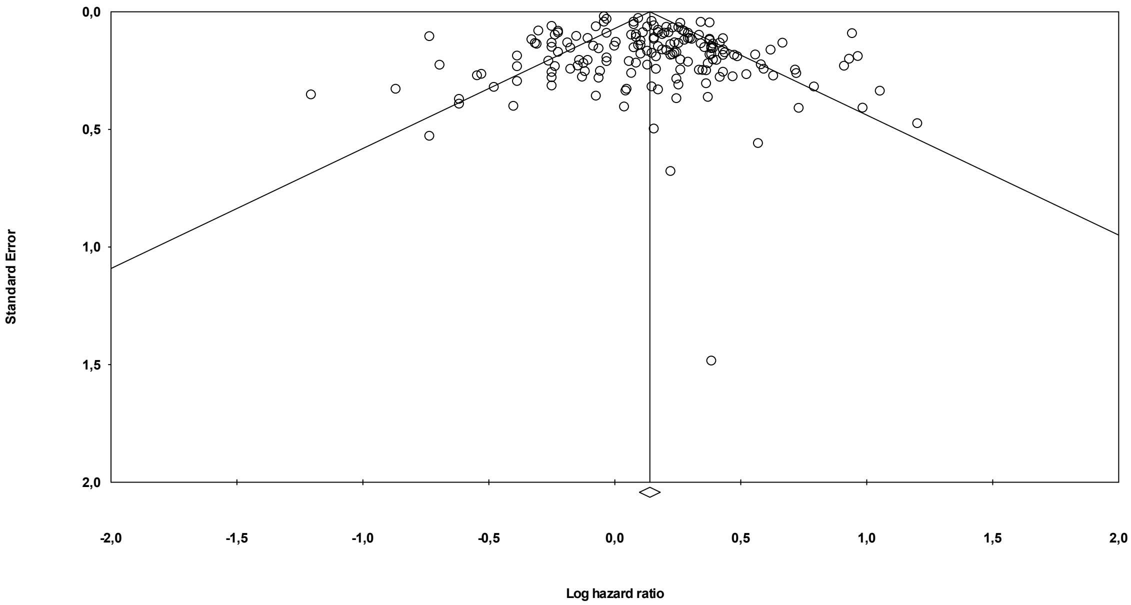


© 2021 Petrelli F et al. *JAMA Network Open.*
